# 3-Bromopyruvate overcomes cetuximab resistance in human colorectal cancer cells by inducing autophagy-dependent ferroptosis

**DOI:** 10.1038/s41417-023-00648-5

**Published:** 2023-08-09

**Authors:** Mingchao Mu, Qin Zhang, Chenye Zhao, Xiaopeng Li, Zilu Chen, Xuejun Sun, Junhui Yu

**Affiliations:** 1 Department of General Surgery, the First Affiliated Hospital of Xi’an Jiaotong University, 710061 Xi’an, Shaanxi China; 2 Department of Dermatology, Northwest Hospital, the Second Affiliated Hospital of Xi’an Jiaotong University, 710004 Xi’an, Shaanxi China

**Keywords:** Chemotherapy, Drug development

## Abstract

Colorectal cancer (CRC) remains a leading cause of cancer-related death worldwide. Cetuximab, in combination with chemotherapy, is effective for treating patients with wild-type KRAS/BRAF metastatic CRC (mCRC). However, intrinsic or acquired drug resistance often limits the use of cetuximab. In this study, we investigated the potential of co-treatment with 3-Bromopyruvate (3-BP) and cetuximab to overcome cetuximab resistance in CRC, both in vitro and in vivo. Our results demonstrated that the co-treatment of 3-BP and cetuximab synergistically induced an antiproliferative effect in both CRC cell lines with intrinsic cetuximab resistance (DLD-1 (KRAS^G13D/-^) and HT29 (BRAF^V600E^)) and in a cetuximab-resistant cell line derived from Caco-2 with acquired resistance (Caco-2-CR). Further analysis revealed that co-treatment induced ferroptosis, autophagy, and apoptosis. Mechanistically, co-treatment inhibited FOXO3a phosphorylation and degradation and activated the FOXO3a/AMPKα/pBeclin1 and FOXO3a/PUMA pathways, leading to the promotion of ferroptosis, autophagy, and apoptosis in DLD-1 (KRAS^G13D/-^), HT29 (BRAF^V600E^), and Caco-2-CR cells. In conclusion, our findings suggest that co-treatment with 3-BP and cetuximab could be a promising strategy to overcome cetuximab resistance in human CRC.

## Introduction

Colorectal cancer (CRC) remains a significant cause of cancer-related mortality worldwide, posing substantial financial burdens and public health challenges [1, 2]. Cetuximab is an effective treatment option for metastatic CRC (mCRC) patients with wild-type KRAS/BRAF genes [3, 4]. However, its efficacy is often limited due to intrinsic or acquired resistance. Mutations in KRAS or BRAF are indicative of poor response to cetuximab treatment, whether used alone or in combination with chemotherapy [5–7]. Even in patients initially responsive to cetuximab, acquired resistance typically develops within 3–12 months of therapy initiation [8]. Despite treatment, the prognosis remains poor for most of these patients.

Ferroptosis is a recently discovered form of programmed, regulated, and autophagy-dependent cell death [9]. Triggering ferroptosis in cancer cells has shown promising effects in cancer therapy by targeting key regulators such as GPX4 and SLC7A11 [10–12], the key regulators of this form of cell death. Novel compounds and drugs that trigger ferroptosis by modulating intracellular antioxidation mechanisms could potentially be used as a therapeutic strategy for treating cancers. 3-Bromopyruvate (3-BP), also known as hexokinase II inhibitor II, has shown promise as an anticancer agent against various types of cancer [13]. 3-BP exerts its anticancer effects by manipulating cell energy metabolism and regulating oxidative stress, as evidenced by the accumulation of reactive oxygen species (ROS) [13–16]. However, it has not been investigated whether 3-BP alone or in combination with other drugs can induce ferroptosis.

Herein, we investigated, in vitro and in vivo, the efficacy of co-treatment of 3-BP with cetuximab to overcome resistance to cetuximab by using different cetuximab-resistant CRC cell lines. We selected three different cell lines to study the efficacy of co-treatment with 3-BP and cetuximab: DLD-1 cells with a KRAS^G13D/-^ mutation, HT29 cells with a BRAF^V600E^ mutation, and Caco-2-CR cells with acquired cetuximab resistance. We revealed that downregulation of the protein level of FOXO3a contributes to resistance to cetuximab in KRAS/BRAF mutant and acquired cetuximab-resistant CRC cells. The co-treatment of 3-BP and cetuximab restores the FOXO3a protein level and its transcriptional activity. This resulted in the activation of the FOXO3a/AMPKα/pBeclin1 pathway and the FOXO3a/PUMA pathway, leading to enhanced ferroptosis, autophagy, and apoptosis. The potentiating cytotoxic effect of the co-treatment effectively allows CRC cells to overcome resistance to cetuximab, highlighting the potential of this combination as a promising strategy.

## Materials and methods

### Reagents

Bromopyruvic acid (S5426), cetuximab (A2000), Compound C (S7840), and U0126 (S1102) were purchased from Selleck (Houston, TX, USA). Deferoxamine (B6068), ferrostatin-1 (A4371), chloroquine (A8628), necrostatin-1 (A4213), and Q-VD-OPh (A8165) were purchased from Apexbio (Boston, MA, USA).

### Cell cultures

HCT116 (KRAS^G13D/-^), HCT116 (KRAS^wt/-^) DLD-1 (KRAS^G13D/-^), DLD-1 (KRAS^wt/-^), RKO (BRAF^V600E/V600E/-^), RKO (BRAF^V600E/-/-^), and RKO (BRAF^wt/-/-^) cell lines were purchased from Horizon Discovery (Cambridge, UK). The Caco-2 cell line was purchased from the Institute of Biochemistry and Cell Biology (Shanghai, China). The HT-29 cell line was purchased from Procell Life Science & Technology Co., Ltd. (Wuhan, China). HCT116, DLD-1, RKO, and HT29 cells were all routinely cultured in Dulbecco’s modified Eagle’s medium (DMEM) (Gibco, NY, USA) supplemented with 10% fetal bovine serum (FBS) (Gibco, NY, USA) at 5% CO_2_ at 37 °C. The Caco-2 cell line was cultured in Minimum Essential Medium (MEM) (Gibco, NY, USA) supplemented with 20% FBS at 5% CO_2_ at 37 °C.

### Lentiviral vector transfection

The lentiviral vector pUBi-EGFP-FOXO3a was purchased from GeneChem Co., Ltd. (Shanghai, China) and used to overexpress FOXO3a. Silencing of BRAF, ATG5, Beclin1, AMPKα, and FOXO3a was achieved through lentiviral transduction using human shRNA purchased from Santa Cruz (CA, USA) specifically targeting BRAF (sc-36368-V), ATG5 (sc-41445-V), Beclin1 (sc-29797-V), AMPKα (sc-45312-V), and FOXO3a (sc-37887-V). The PUMA CRISPR plasmid (sc-400464-KO-2), ERK1 siRNA (sc-29307), and ERK2 siRNA (sc-35335) were also purchased from Santa Cruz (Santa Cruz, CA, USA). All transfections were performed following the manufacturer’s protocol.

### Cell viability assay

Cell viability was determined by a Cell Counting Kit 8 (Dojindo, Japan). Cells were seeded in a 96-well plate and incubated. At the indicated time point, 10 μl CCK-8 solution was added to each well containing 100 µl medium. Following a 3-hour incubation, absorbance was measured at a wavelength of 450 nm to determine cell viability.

### Malondialdehyde (MDA) assay

The MDA level was measured by a Lipid Peroxidation MDA Assay Kit (S0131S, Beyotime, Shanghai, China) according to the manufacturer’s protocol.

### Lipid reactive oxygen species (ROS) level assay

The ROS level was measured using a Reactive Oxygen Species Assay Kit (S0033M, Beyotime, Shanghai, China) according to the manufacturer’s protocol.

### Intracellular iron assay

The intracellular iron level was measured by an Iron Assay Kit (ab83366, Abcam, UK) according to the manufacturer’s protocol. The intracellular iron level was also assessed by using FerroOrange (F374-10, Dojindo, Japan). The culture medium was replaced by HBSS supplied with 10 μg/ml Hoechst 33342 (B8040, Solarbio, Beijing, China) for 30 min. Then, the supernatant was discarded, and the cells were washed with HBSS three times. HBSS supplied with 1 μmol/L FerroOrange was added, and the cells were incubated at 37 °C and 5% CO_2_ for 30 min. The cells were observed under a fluorescence microscope without washing.

### Colony formation assay

For the colony formation assay, 500 DLD-1 (KRAS^G13D/-^) cells, 500 Caco-2-CR cells, or 1000 HT29 (BRAF^V600E^) cells were seeded in each well of six-well plates and cultured for two weeks. Colonies consisting of more than 50 cells were counted.

### Glutamate release assays

The Glutamate-Glo Assay kit (Promega, Madison, WI, USA) was used to quantify the amount of glutamate released into the conditioned medium. Cells (5 × 10^3^) were plated in a 96-well plate in DMEM (A14430, Gibco, NY, USA) containing 5 mM glucose, 2 mM glutamine, and 10–20% FBS. After the indicated treatment for 24 h, 10 µl of the medium was diluted in 90 µl of PBS. Fifty microliters of the sample was transferred to a 96-well plate and mixed with 50 μl of luciferin detection solution following the manufacturer’s protocol. The plate was incubated at room temperature for 1 h, and luminescence was measured with a microplate reader (BioTek Cytation 5, VT, USA). The glutamate levels were normalized to the total cell number, which was determined by MTT assays at the end of the experiment.

### GSH assay

The relative GSH concentration in tissue lysates was assessed using a Glutathione Microplate Assay Kit (abs580006, Absin, Shanghai, China) according to the manufacturer’s instructions.

### Total protein extraction and western blotting analysis

Cell lysates were heat denatured, separated by SDS‒PAGE, transferred to PVDF membranes (abs931, Absin, Shanghai, China), and blocked with 5% skimmed milk. Then, membranes were incubated, respectively with anti-BRAF (1:1000, ab33899), anti-GPX4 (1:1000, ab125066), anti-PUMA (1:1000, ab9643), anti-LC3B (1:2000, ab192890), anti-MLKL (1:500, ab184718), anti-phospho-MLKL (1:1000, ab196436), anti-ERK1/2 (1:1000, ab184699), anti-Ubiquitin (1:1000, ab134953), anti-AMPKα (1:2000, ab32047), anti-p62 (1:1000, ab109012), anti-Beclin1 (1:2000, ab207612) primary antibodies from Abcam (UK), anti-Alpha Tubulin (1:1000, 11224-1-AP), anti-FOXO3a (1:1000, 10849-1-AP), anti- SLC7A11 (1:1000, 26864-1-AP), ati-H3 (1:1000, 17168-1-AP) primary antibodies from Proteintech (IL, USA), anti-Cleaved Caspase-3 (1:1000, #9661), anti-phospho-Beclin1 (1:1000, #14717), anti-phosphor-AMPKα (1:1000, #2535), anti-phospho-FOXO3a (1:1000, #64616), anti-phospho-ERK1/2 (1:1000, #4370) primary antibodies from Cell Signaling Technology (MA, USA) and anti-ATG5 (1:1000, ET1611-38) from HUABIO (Hangzhou, China) overnight at 4 °C and anti-rabbit (1:5000, ab205718, absin, Shanghai, China) or anti-mouse (1:5000, abs20039, absin, Shanghai, China) secondary antibodies for 1 h at room temperature. Immunoblots were visualized using an ECL detection reagent (BMU101-CN, Abbkine Scientific, Wuhan, China).

### Isolation of nuclear and cytoplasmic proteins

Nuclear and cytoplasmic proteins were separated by a Nuclear Cytoplasmic Isolation Kit (P0028, Beyotime, Shanghai, China) according to the manufacturer’s protocol.

### Immunoprecipitation assay

Cell lysis was performed using IP lysis buffer (Beyotime, Shanghai, China). The lysate was incubated with 5 μg of primary antibody at 4 °C overnight, followed by the addition of magnetic beads (Thermo Scientific, 88805) for 15 min. The beads were collected using a magnetic stand, and the supernatant was discarded. The beads were washed three times, and then low-pH elution buffer was added, followed by incubation at room temperature for 5 min. The elution was neutralized by adding neutralization buffer, and the resulting eluate was used for western blotting analysis.

### Cell apoptosis assay

Apoptotic cell death was assessed using an apoptosis detection kit (KGA1023, KeygenTec, Nanjing, China) according to the manufacturer’s instructions.

### Luciferase reporter assay

The pGL3-AMPKα (full-length promoter) plasmid and pRLK-TK plasmid were prepared by GeneChem Co., Ltd. (Shanghai, China). The pFOXO3-TA-Luc plasmid designed to measure the transcriptional activity of FOXO3a was purchased from Beyotime (Shanghai, China). 10^4^ cells were seeded on a 96-well plate. Then, 100 ng of firefly luciferase vector was transfected along with 10 ng of pRLK-TK vector using Lipo3000 (Invitrogen, MA, USA). After 48 h of transfection, luminescence was detected using the Dual-Luciferase Reporter Gene Assay Kit II (Beyotime, Shanghai, China) and Varioskan Flash (Thermo Scientific, MA, USA).

### Xenograft tumor models

The experiment was approved by the First Affiliated Hospital of Xi’an Jiaotong University Experimental Animal Ethics Committee. For each model, we subcutaneously transplanted 1 × 10^6^ cells with 0.1 ml PBS into the right dorsal flanks of 8-week-old female nude mice. Eight days postinoculation, the tumor-bearing mice were separated randomly into four groups of five mice each. The treatment was initiated by intraperitoneal drug administration every 4 days. Mice were sacrificed 28 days after inoculation. Tumors were measured with a caliper every 4 days, and volume was calculated as ½(L × W^2^).

### Statistical analysis

Data are presented as the mean ± SD. The differences among the groups were compared by two-sided Student’s t-test or one-way ANOVA. Correlations were analyzed by using Pearson linear regression analysis. All statistics were calculated by R Project (Version 4.0.3). A *P* value < 0.05 indicates statistical significance. All data were generated from three independent experiments.

## Results

### The co-treatment of 3-BP with cetuximab improved the cytotoxic effect on cetuximab-resistant human CRC cell lines

**Fig. 1:**
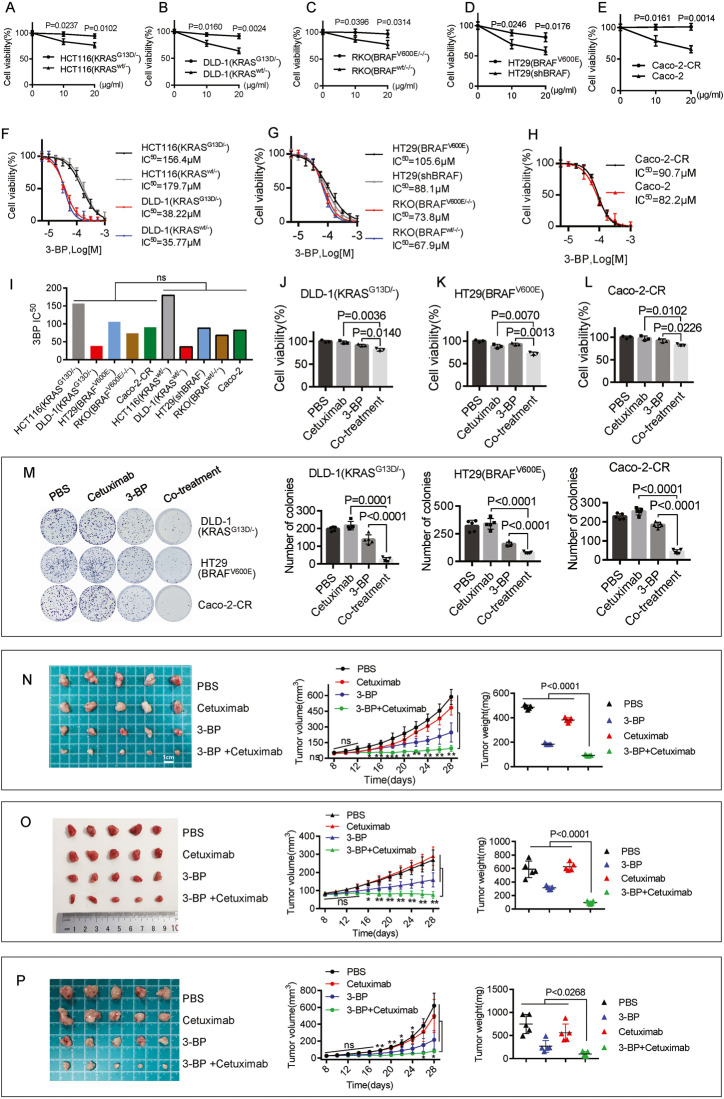
The co-treatment of 3-BP with cetuximab improved the cytotoxic effect. **A**–**E** The cells were treated with cetuximab at 10 or 20 μg/ml cetuximab for four days. **A** The response of HCT116 (KRAS^G13D/-^) and HCT116 (KRAS^wt/-^) cells to cetuximab. **B** The response of DLD-1(KRAS^G13D/-^) and DLD-1(KRAS^wt/-^) cells to cetuximab. **C** The response of RKO (BRAF^V600E/-/-^) and RKO (BRAF^wt/-/-^) cells to cetuximab. **D** The response of HT29(BRAF^V600E^) cells transfected with or without shRNA targeting BRAF to cetuximab. **E** The response of Caco-2-CR and Caco-2 cells to cetuximab. **F** The dose-response curve and IC^50^ of 3-BP in HCT116 (KRAS^G13D/-^), HCT116 (KRAS^wt/-^), DLD-1(KRAS^G13D/-^) and DLD-1 (KRAS^wt/-^) cells. **G** The dose-response curve and IC^50^ of 3-BP in RKO (BRAF^V600E/-/-^), RKO (BRAF^wt/-/-^), HT29(BRAF^V600E^), and HT29(shBRAF) cells. **H** The dose-response curve and IC^50^ of 3-BP in Caco-2 and Caco-2-CR cells. **I** The Comparison of the IC^50^ values between cetuximab-resistant cell lines and their corresponding cetuximab-sensitive cell lines. (**J**–**L**) The cell viability after the treatment of 5 μM 3-BP and/or 10 μg/ml cetuximab for four days in DLD-1(KRAS^G13D/-^) cells (**J**), HT29(BRAF^V600E^) cells (**K**) and Caco-2-CR cells (**L**). **M** The cells were exposed to 5 μM 3-BP and/or 10 μg/ml cetuximab for two weeks, and the colony formation was evaluated. **N**–**P** The xenograft nude mouse models, DLD-1(KRAS^G13D/-^) (**N**), HT29(BRAF^V600E^) (**O**), and Caco-2-CR (**P**), were established. The tumor-bearing mice were treated with PBS (0.2 ml), 3-BP (4 mg/kg/day, dissolved in 0.2 ml PBS), cetuximab (50 mg/kg/day, dissolved in 0.2 ml PBS), or the co-treatment of both 3-BP and cetuximab. The volumes and tumor weights were evaluated. Data are expressed as mean ± SD, *n* = 3 biological replicates in (**A**–**H**), (**J**–**L**), and *n* = 5 biological replicates in (**M**–**P**). *P < 0.05, **P < 0.01, ns: not significant.

We initially conducted a comparison of the response to cetuximab between KRAS/BRAF-mutant CRC cell lines and their corresponding parent wild-type cells. These cells were treated with cetuximab at 10 or 20 μg/ml for 4 days. As expected, KRAS mutation (KRAS^G13D^) conferred significantly higher cetuximab resistance in HCT116 and DLD-1 cells (Fig. 1A). BRAF mutation (BRAF^V600E^) conferred significantly higher cetuximab resistance in RKO cells (Fig. 1C). Knockdown of BRAF gene expression (Supplementary Figure 1A) sensitized HT29 (BRAF^V600E^) cells to cetuximab treatment (Fig. 1D). Then, we generated an acquired cetuximab-resistant cell line, Caco-2-CR, which could be cultured in medium supplemented with 200 μg/ml cetuximab by chronically exposing Caco-2 cells to increasing concentrations of cetuximab for more than six months (Fig. 1E).

Next, we compared the responses to 3-BP (Supplementary Figure 1B) in these CRC cell lines. 3-BP showed a different inhibitory effect on the proliferation of these cell lines, with IC^50^ values at 24 h ranging from 35.77 μM to 179.7 μM (Fig. 1F–H). There was no significant difference in the cytotoxic effects of 3-BP between cetuximab-resistant cell lines (HCT116(KRAS^G13D/-^), DLD-1(KRAS^G13D/-^), HT29(BRAF^V600E^), RKO(BRAF^V600E/-/-^), Caco-2-CR) and their paired cetuximab-sensitive cell lines (HCT116(KRAS^wt/-^), DLD-1(KRAS^wt/-^), HT29(shBRAF), RKO(BRAF^wt/-/-^), Caco-2), indicating that the cytotoxic effect of 3-BP in these CRC cell lines is independent of their response to cetuximab.

Next, two cell lines, DLD-1 (KRAS^G13D/-^) and HT29 (BRAF^V600E^), were used as models of intrinsic resistance to cetuximab, and Caco-2-CR was used as a model of acquired resistance to cetuximab for further study. We then treated these cell lines with 3-BP and/or cetuximab at the indicated concentrations (Supplementary Figure 1C, D, E). A synergistic effect was observed in cell inhibition percentages greater than 21.5% in DLD-1(KRAS^G13D/-^) and 17.6% in Caco-2-CR cells. The synergistic effect in HT29 (BRAF^V600E^) cells was calculated using CompuSyn software (Supplementary Figure 1F). Then, we treated cells with 5 μM 3-BP and/or 10 μg/ml cetuximab for four days and observed that co-treatment of cetuximab with 3-BP showed stronger antiproliferative activity than cetuximab or 3-BP as a single agent (Fig. 1J, K, L). The colony formation assay was also performed to determine the effect of the combination. The cells were treated with 5 μM 3-BP and/or 10 μg/ml cetuximab for two weeks. As a result, co-treatment showed stronger suppression of colony formation than either of the single drugs (Fig. 1M).

To further investigate the antiproliferative efficacy in vivo, three xenograft nude mouse models (DLD-1(KRAS^G13D/-^), HT29(BRAF^V600E^), and Caco-2-CR) were established. Eight days postinoculation, xenografted mice were randomly divided into four groups and intraperitoneally injected with PBS, 3-BP, cetuximab, or a combination of 3-BP and cetuximab every four days for five injections. Both treatments with 3-BP alone or co-treatment with 3-BP and cetuximab significantly reduced the tumor volume and tumor weight on Day 28, but co-treatment showed a greater reduction than 3-BP alone (Fig. 1N–P).

### Co-treatment of 3-BP with cetuximab induces autophagy, ferroptosis, and apoptosis in cetuximab-resistant human CRC cell lines

**Fig. 2:**
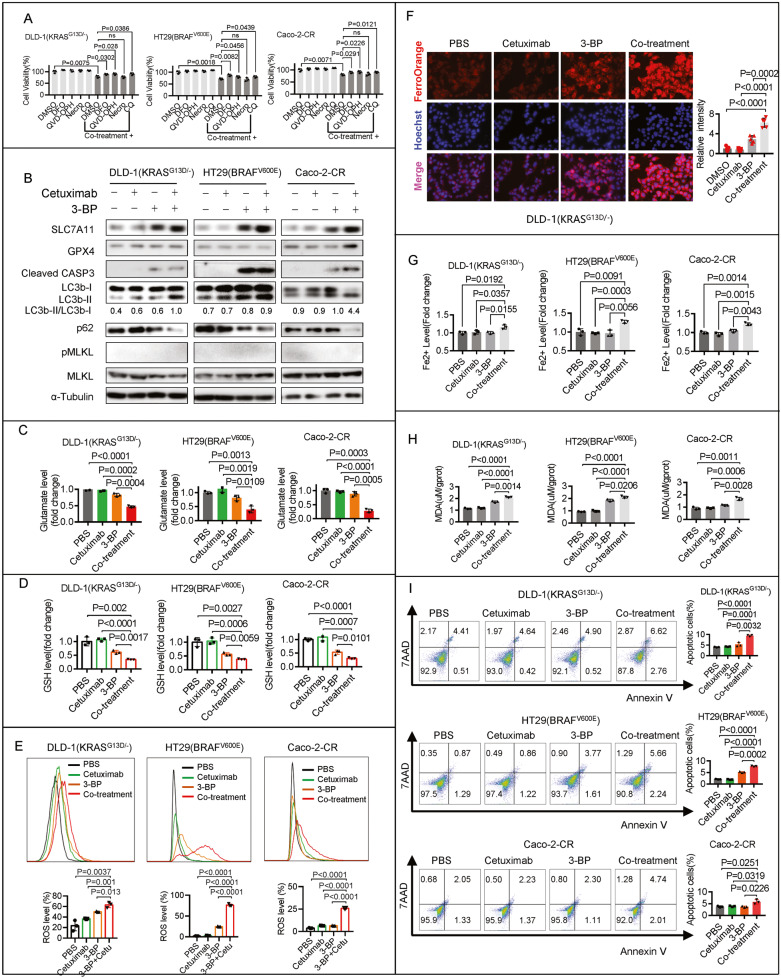
Co-treatment of 3-BP with cetuximab induces autophagy, ferroptosis, and apoptosis in cetuximab-resistant human CRC cell lines. **A** The effects of co-treatment with 5 μM 3-BP and 10 μg/ml cetuximab for four days were examined in DLD-1(KRAS^G13D/-^), HT29(BRAF^V600E^), or Caco-2-CR cells. DFO (100 μM), QVD-OPH (25 μM), Nec-1 (25 μM), or CQ (50 μM) were used to determine their impact on cell response. **B** DLD-1(KRAS^G13D/-^), HT29(BRAF^V600E^), or Caco-2-CR cells were treated with 5 μM 3-BP and/or 10 μg/ml cetuximab for four days, and immunoblotting assay analyzed the protein level of SLC7A11, GPX4, Cleaved Caspase-3, LC3b, p62, MLKL, and phosphorylated-MLKL. **C** The glutamate release after the cells were treated with 5 μM 3-BP and/or 10 μg/ml cetuximab for 24 hours. **D**–**I** The cells were treated with 5 μM 3-BP and/or 10 μg/ml cetuximab for four days, and the GSH level (**D**), the ROS level (**E**), the FerroOrange visualized Fe^2+^ level (**F**), the colorimetric method determined Fe^2+^ level (**G**), the MDA production (**H**) and the apoptotic cells (**I**) were evaluated. Data are expressed as mean ± SD, *n* = 3 biological replicates in (**A**), (**C**), (**D**), (**E**), (**G**), (**H**), (**I**), and *n* = 5 biological replicates in (**F**). ns: not significant.

To investigate the mechanism by which co-treatment contributes to cell death, we exposed the cells to a panel of inhibitors of various cell death pathways. An inhibitor of RIPK1 (necrostatin-1, Nec-1) did not prevent the cell death induced by co-treatment, while inhibitors of caspase (QVD-OPH), autophagy (chloroquine, CQ), and iron chelation (deferoxamine, DFO) significantly prevented cell death (Fig. 2A), indicating that multiple mechanisms contribute to cell death. A combination of different cell death inhibitors completely inhibited the anticancer activity of the co-treatment in Caco-2-CR cells but not in DLD-1(KRAS^G13D/-^) or HT29(BRAF^V600E^) cells (Supplementary Figure 2A).

To gain a complete understanding of the cell death mechanism, we assayed the key ferroptosis regulators SLC7A11 and GPX4, as well as key biomarkers of apoptosis (caspase-3), necroptosis (MLKL), and autophagy (LC3), in parallel. Immunoblotting analysis showed that the protein level of GPX4 was not affected in DLD-1 (KRAS^G13D/-^) and HT29 (BRAF^V600E^) cells but was upregulated in Caco-2-CR cells (Fig. 2B). The protein level of SLC7A11 was significantly upregulated in all three cell lines following co-treatment (Fig. 2B). In contrast, a glutamate release assay indicated that the activity of system Xc^−^ was significantly inhibited following co-treatment (Fig. 2C). Further functional assays showed that co-treatment with 3-BP and cetuximab led to glutathione (GSH) depletion (Fig. 2D), reactive oxygen species (ROS) production (Fig. 2E), Fe^2+^ accumulation (Fig. 2F, G, Supplementary Figure 2B), and malondialdehyde (MDA) production (Fig. 2H) in DLD-1 (KRAS^G13D/-^), HT29 (BRAF^V600E^), and Caco-2-CR cells. Immunoblotting analysis showed that the autophagy marker LC3-II/LC3-I ratio increased and p62 degraded following co-treatment (Fig. 2B). The level of the apoptotic marker cleaved caspase-3 was also significantly increased following co-treatment. Flow cytometry confirmed that apoptosis was induced by co-treatment (Fig. 2I). The necroptotic marker phosphorylated MLKL (pMLKL) was not detected following co-treatment (Fig. 2B). These results suggest that co-treatment with 3-BP and cetuximab induces ferroptosis, autophagy, and apoptosis but not necroptosis in cetuximab-resistant human CRC cell lines.

As ferroptosis is recognized as a type of autophagy-dependent cell death, we tested whether the knockdown of ATG5 or Beclin1 affected 3-BP/cetuximab-induced ferroptosis. ATG5 knockdown attenuated Fe^2+^ accumulation but did not affect glutamate release or GSH levels (Supplementary Figure 2D, E, F). Beclin1 knockdown reactivated glutamate release and restored GSH but did not affect Fe^2+^ levels (Supplementary Figure 2D, E, F). Knockdown of either ATG5 or Beclin1 attenuated the cell death and MDA production induced by co-treatment (Supplementary Figure 2D, E). These results suggest that autophagy-dependent ferroptosis is induced by co-treatment with 3-BP and cetuximab in DLD-1 (KRAS^G13D/-^), HT29 (BRAF^V600E^), and Caco-2-CR cells.

### Co-treatment with 3-BP and cetuximab inhibits system Xc^−^ activity by activating the FOXO3a/AMPKα/pBeclin1 pathway

**Fig. 3:**
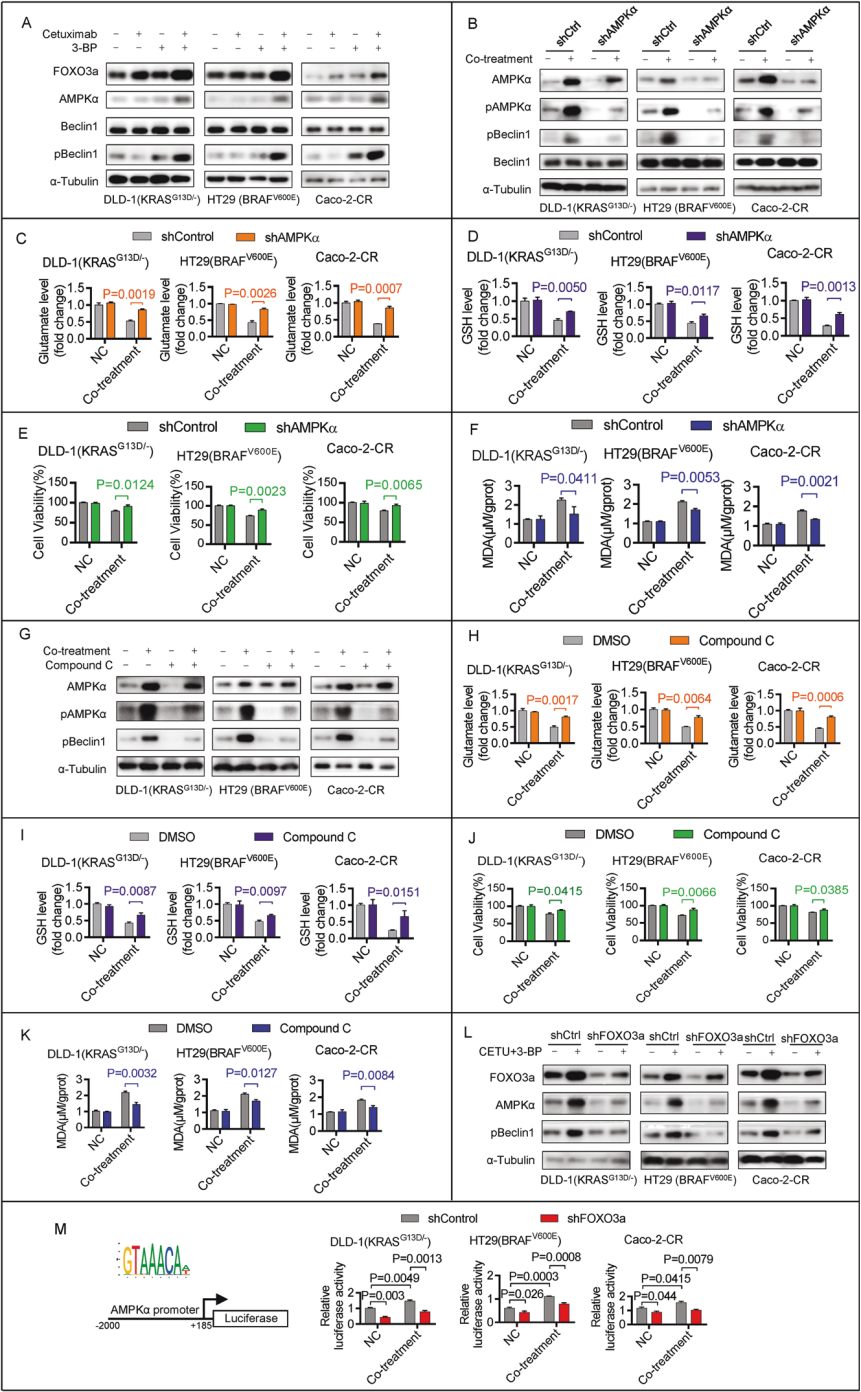
Co-treatment of 3-BP and cetuximab inhibits system Xc^−^ activity by activating FOXO3a/AMPKα/pBeclin1 pathway. **A** DLD-1(KRAS^G13D/-^), HT29(BRAF^V600E^), or Caco-2-CR cells were treated with 5 μM 3-BP and/or 10 μg/ml cetuximab for four days, and immunoblotting assay analyzed the protein level of FOXO3a, AMPKα, phosphorylated Beclin1 and total Beclin1. **B**–**F** DLD-1(KRAS^G13D/-^), HT29(BRAF^V600E^), or Caco-2-CR cells with or without AMPKα knockdown were treated with 5 μM 3-BP and 10 μg/ml cetuximab for four days. Immunoblotting assay analyzed the protein level of AMPKα, phosphorylated AMPKα, total Beclin1, and phosphorylated Beclin1 (**B**). The effect of AMPKα knockdown on glutamate released (**C**), GSH level (**D**), cell viability (**E**), and MDA production (**F**) was evaluated. **G**–**K** DLD-1(KRAS^G13D/-^), HT29(BRAF^V600E^), or Caco-2-CR cells with or without Compound C (1 μM) treatment were treated with 5 μM 3-BP and 10 μg/ml cetuximab for four days. Immunoblotting assay analyzed the protein level of total AMPKα, phosphorylated AMPKα, total Beclin1, and phosphorylated AMPKα (**G**). The effect of Compound C (1 μM) treatment on glutamate released (**H**), GSH level (**I**), cell viability (**J**), and MDA production (**K**) was evaluated. **L** DLD-1(KRAS^G13D/-^), HT29(BRAF^V600E^), or Caco-2-CR cells with or without FOXO3a knockdown were treated with 5 μM 3-BP and 10 μg/ml cetuximab for four days. Immunoblotting assay analyzed the protein level of FOXO3a, AMPKα, and phosphorylated Beclin1. **M** The DLD-1(KRAS^G13D/-^), HT29(BRAF^V600E^), or Caco-2-CR cells with or without FOXO3a knockdown were treated with 5 μM 3-BP and 10 μg/ml cetuximab for 48 h. The effect of FOXO3a knockdown on the transcriptional activity of the AMPKα gene was evaluated. Data are expressed as mean ± SD, *n* = 3 biological replicates.

Next, we investigated the inhibition mechanism of system Xc^−^ in the co-treatment. A previous study reported that AMPKα-mediated Beclin1 phosphorylation directly blocked System Xc^−^ Activity and contributed to ferroptosis [17], and FOXO3a was able to activate the transcription of AMPKα [18]. We then investigated whether the FOXO3a/AMPKα/pBeclin1 pathway mediated system Xc^−^ activity inhibition by co-treatment. Immunoblotting analysis showed that the protein levels of FOXO3a, AMPKα, and phosphorylated Beclin1 (pBeclin1) but not Beclin1 were upregulated after co-treatment (Fig. 3A). Decreased phosphorylated AMPKα (pAMPKα) by knockdown AMPKα gene expression attenuated the co-treatment-induced pBeclin1 protein upregulation but did not affect Beclin1 protein levels (Fig. 3B). Further functional assays showed that AMPKα knockdown reactivated glutamate release (Fig. 3C), restored GSH (Fig. 3D), and attenuated cytotoxicity (Fig. 3E) and MDA production (Fig. 3F) induced by co-treatment. Compound C inhibited AMPKα phosphorylation and attenuated co-treatment-induced pBeclin1 protein upregulation (Fig. 3G). Functional assays showed that Compound C, similar to AMPKα knockdown, reactivated glutamate release (Fig. 3H), restored GSH (Fig. 3I), and attenuated cytotoxicity (Fig. 3J) and MDA production (Fig. 3K) induced by co-treatment. These results suggest that pAMPKα-mediated Beclin1 phosphorylation contributed to system Xc^−^ activity inhibition in the co-treatment. Additionally, FOXO3a knockdown attenuated the co-treatment-induced upregulation of AMPKα and pBeclin1 (Fig. 3L). Next, we generated luciferase reporter plasmids by introducing the promoter region (−2000 bp to +185 bp) of the human AMPKα gene into the pGL3-luciferase reporter plasmid (Fig. 3M). Luciferase activity was significantly increased following co-treatment, and this increase was attenuated following FOXO3a knockdown (Fig. 3M). This result suggests that FOXO3a was able to activate the transcription of AMPKα. Together, these data suggest that activation of the FOXO3a/AMPKα/pBeclin1 pathway contributes to the cell death induced by co-treatment with 3-BP and cetuximab in cetuximab-resistant human CRC cell lines.

### Co-treatment with 3-BP and cetuximab stabilizes FOXO3a by inhibiting FOXO3a phosphorylation

**Fig. 4:**
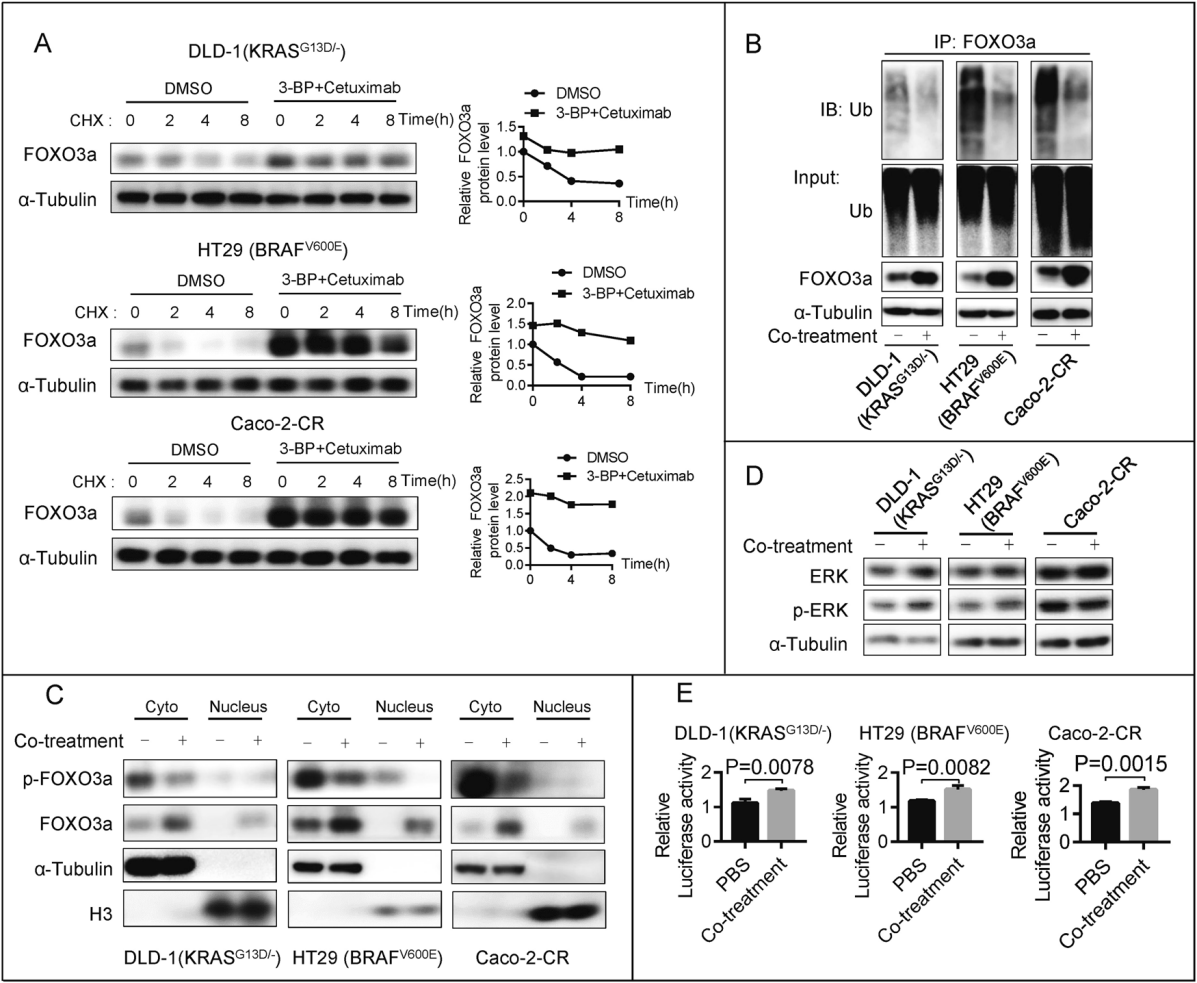
Co-treatment of 3-BP and cetuximab stabilizes FOXO3a via inhibiting FOXO3a phosphorylation. **A** The protein levels of FOXO3a were examined in DLD-1 (KRAS^G13D/-^), HT29 (BRAFV^600E^), and Caco-2-CR cells after co-treatment with 5 μM 3-BP and 10 μg/ml cetuximab, followed by treatment with cycloheximide (CHX, 50 μg/ml) for different durations. **B** The anti-FOXO3a immunoprecipitation was prepared following the co-treatment, then it was probed for the level of ubiquitination using the antibody specifically against ubiquitin. **C** The phosphorylated FOXO3a and total FOXO3a protein levels in the cytoplasm and nucleus were examined following the co-treatment. **D** The phosphorylated ERK and total ERK protein levels were examined following the co-treatment. **E** The luciferase activity levels were measured in DLD-1 (KRAS^G13D/-^) cells, HT29 (BRAF^V600E^) cells, and Caco-2-CR cells that were co-transfected with pFOXO3-TA-Luc and pRLK-TK plasmids, after being co-treated for 48 h. The relative luciferase activities were then evaluated to determine the effects of the co-treatment. Data are expressed as mean ± SD, *n* = 3 biological replicates.

We then investigated the effects of co-treatment on preventing FOXO3a degradation. A cycloheximide (CHX) chase experiment showed that co-treatment rescued the FOXO3a protein level and resulted in a steady level of FOXO3a protein (Fig. 4A). Next, we confirmed that co-treatment promoted the deubiquitination of the FOXO3a protein (Fig. 4B). A previous study reported that ERK could directly phosphorylate FOXO3a and phosphorylated FOXO3a (pFOXO3a) degraded via the ubiquitin-proteasome pathway [19]. Following co-treatment with 3-BP and cetuximab, we found that pFOXO3a was downregulated (Fig. 4C), indicating that co-treatment inhibits FOXO3a phosphorylation. However, the protein levels of ERK and phosphorylated ERK (pERK) (Fig. 4D) were not affected by co-treatment, indicating that co-treatment induced ERK-independent FOXO3a phosphorylation inhibition. These results suggest that co-treatment with 3-BP and cetuximab suppresses FOXO3a phosphorylation and inhibits FOXO3a degradation. Importantly, co-treatment promoted FOXO3a nuclear accumulation (Fig. 4C) and enhanced its transcriptional activity (Fig. 4E).

### Co-treatment of 3-BP with cetuximab induces autophagy and apoptosis by inducing FOXO3a stabilization in cetuximab-resistant human CRC cell lines

A previous study reported that FOXO3a was a trigger for apoptosis through the upregulation of genes for cell death, such as PUMA [20]. Next, we investigated whether co-treatment induced apoptosis by activating the FOXO3a/PUMA pathway. Immunoblotting analysis showed that PUMA protein levels were upregulated by co-treatment with 3-BP and cetuximab, and co-treatment-induced PUMA expression was abolished by FOXO3a knockdown (Supplementary Figure 3A). PUMA knockout abrogated co-treatment-induced caspase-3 activation (Supplementary Figure 3B). Flow cytometry further confirmed that FOXO3a mediates co-treatment-induced apoptosis (Supplementary Figure 3C). These results suggest that co-treatment with 3-BP and cetuximab induces apoptosis by activating the FOXO3a/PUMA pathway.

FOXO3a is a transcriptional regulator reported to be linked to autophagy [21]. It can translocate from the cytoplasm to the nucleus to activate the transcription of a subset of autophagy-related genes, promoting autophagy [22, 23]. We then investigated the role of FOXO3a in co-treatment-induced autophagy. Immunoblotting analysis showed that FOXO3a knockdown effectively attenuated the co-treatment-induced LC3-II/LC3-I ratio increase and p62 degradation (Supplementary Figure 3D). The result suggests that the co-treatment of 3-BP and cetuximab induces autophagy-dependent on FOXO3a stabilization.

### Co-treatment of 3-BP with cetuximab induces ferroptosis, autophagy, and apoptosis in xenograft nude mouse models

**Fig. 5:**
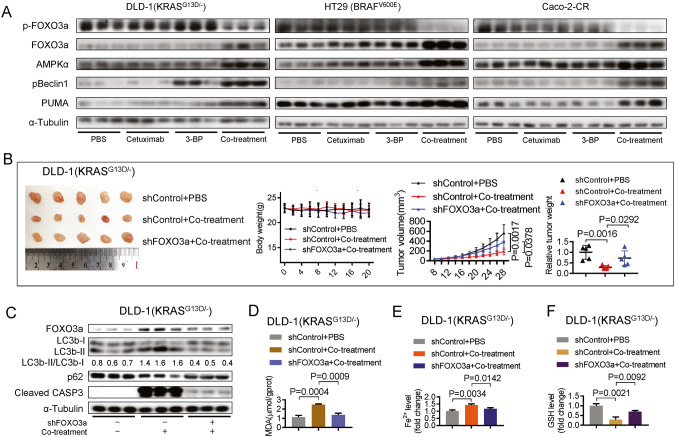
Co-treatment of 3-BP with cetuximab induces ferroptosis, autophagy, and apoptosis in xenograft nude mouse models. **A** Immunoblotting analysis to evaluate the protein levels of phosphorylated FOXO3a, total FOXO3a, AMPKα, pBeclin1, and PUMA in tumor xenografts in nude mice after treatment with PBS (0.2 ml), 3-BP (4 mg/kg/day, dissolved in 0.2 ml PBS), cetuximab (50 mg/kg/day, dissolved in 0.2 ml PBS), or a combination of both 3-BP and cetuximab. **B** The xenograft nude mouse models were established using DLD-1(KRAS^G13D/-^) with or without FOXO3a knockdown. The body weights, tumor volumes, and tumor weights were evaluated following the treatment of PBS (0.2 ml), or a combination of 3-BP (2 mg/kg/day, dissolved in 0.2 ml PBS) and cetuximab (25 mg/kg/day, dissolved in 0.2 ml PBS). **C** Immunoblotting analyzed protein levels of LC3b, p62, Cleaved Caspase-3, and FOXO3a in tumor xenografts. **D**–**F** The effect of FOXO3a knockdown on the co-treatment-induced MDA production (**D**), Fe^2+^ accumulation (**E**), and GSH depletion (**F**) in tumor xenografts were evaluated. Data are expressed as mean ± SD, *n* = 5 biological replicates. ns: not significant.

To investigate whether FOXO3a plays a similar role in vivo as it does in vitro, we conducted immunoblotting and found that co-treatment induced depletion of pFOXO3a protein but accumulation of AMPKα, pBeclin1, PUMA, and total FOXO3a proteins (Fig. 5A). The changes were consistent with the in vitro data.

Next, we established xenograft nude mouse models with or without FOXO3a knockdown. We observed a significant reduction in tumor size and weight in the group without FOXO3a knockdown compared to the group with FOXO3a knockdown in DLD-1 (KRAS^G13D/-^) xenograft nude mouse models (Fig. 5B). This effect was also observed in HT29 (BRAF^V600E^) (Supplementary Figure 4A) and Caco-2-CR (Supplementary Figure 4F) xenograft nude mouse models. Immunoblotting analysis showed that FOXO3a knockdown abolished the co-treatment-induced increase in the LC3-II/LC3-I ratio, degradation of p62, and activation of caspase-3 (Fig. 5C, Supplementary Figure 4B, G). Functional assays showed that FOXO3a knockdown attenuated co-treatment-induced MDA production (Fig. 5D, Supplementary Figure 4C, H), Fe^2+^ accumulation (Fig. 5E, Supplementary Figure 4D, I), and GSH depletion (Fig. 5F, Supplementary Figure 4E, J). Our results revealed that FOXO3a knockdown attenuates co-treatment-induced ferroptosis, autophagy, and apoptosis in xenograft nude mouse models, indicating that FOXO3a plays an important role in mediating the anticancer effect of co-treatment in vivo.

### The FOXO3a protein level is downregulated in cetuximab-resistant CRC cell lines

**Fig. 6:**
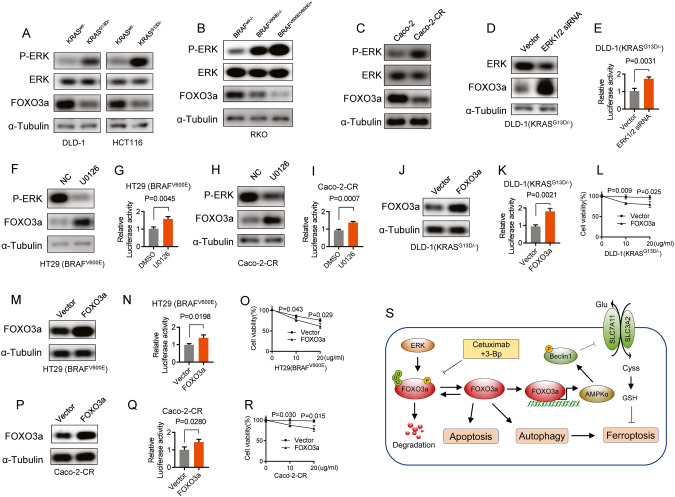
The FOXO3a protein level is downregulated in cetuximab-resistant CRC cell lines. **A** KRAS^G13D^ mutation led to an increase in ERK phosphorylation and a decrease in FOXO3a protein levels in HCT116 and DLD-1 isogenic cells. **B** In RKO isogenic cells, the BRAF^V600E^ mutation caused elevated phosphorylation of ERK and reduced FOXO3a protein levels. **C** The protein levels of FOXO3a, phosphorylated ERK, and total ERK were examined in Caco-2 cells and Caco-2-CR cells. **D**, **E** DLD-1(KRAS^G13D/-^) cells were transfected with or without siRNAs targeting ERK1/2, then FOXO3a and ERK protein levels were analyzed (**D**). Cells were co-transfected with pFOXO3-TA-Luc and pRLK-TK plasmids to evaluate FOXO3a reporter activity (**E**). **F**–**I** FOXO3a and phosphorylated ERK protein levels were assessed in HT29 (BRAF^V600E^) cells (**F**) and Caco-2-CR cells (**H**) following treatment with U0126 (2 μM) for 4 h. FOXO3a reporter activity was examined in HT29 (BRAF^V600E^) cells (**G**) and Caco-2-CR cells (**I**) with or without U0126 treatment. **J**–**L** FOXO3a was overexpressed in DLD-1(KRAS^G13D/-^) cells (**J**), and the activity of the FOXO3a reporter (**K**) and response to cetuximab (**L**) were evaluated in DLD-1(KRAS^G13D/-^) cells with or without FOXO3a overexpression. **M**–**O** FOXO3a was overexpressed in HT29 (BRAF^V600E^) cells (**M**), and the activity of the FOXO3a reporter (**N**) and response to cetuximab (**O**) were evaluated in HT29 (BRAF^V600E^) cells with or without FOXO3a overexpression. **P**–**R** FOXO3a was overexpressed in Caco-2-CR cells (**P**), and the activity of the FOXO3a reporter (**Q**) and response to cetuximab (**R**) were evaluated in Caco-2-CR cells with or without FOXO3a overexpression. **S** Schematic diagrams illustrating how FOXO3a mediates 3-BP/cetuximab co-treatment-induced apoptosis and autophagy-dependent ferroptosis in cetuximab-resistant CRC cells.

Next, we investigated how KRAS/BRAF mutations affect FOXO3a activity and how FOXO3a is regulated in CRC cells with acquired cetuximab resistance. Immunoblotting analysis revealed a significant decrease in the protein level of FOXO3a in HCT116 and DLD-1 cells with the KRAS^G13D^ mutation compared to their wild-type KRAS parent cells (Fig. 6A). Additionally, FOXO3a protein was observed to be downregulated in RKO cells with the BRAF^V600E^ mutation compared to their wild-type BRAF parent cells (Fig. 6B). Similarly, the FOXO3a protein was also found to be downregulated in Caco-2-CR cells in comparison to Caco-2 cells (Fig. 6C). A previous study reported that ERK phosphorylated FOXO3a and enhanced FOXO3a degradation through the ubiquitin-proteasome pathway [19]. Immunoblotting analysis revealed that phosphorylated ERK, but not total ERK, was upregulated in DLD-1 (KRAS^G13D/-^), HCT116 (KRAS^G13D/-^), and RKO (BRAF^V600E^) cells in comparison to their respective wild-type parent cells (Fig. 6A–C). ERK knockdown rescued FOXO3a protein levels (Fig. 6D) and increased its transcriptional activity (Fig. 6E) in DLD-1 (KRAS^G13D/-^). Furthermore, inhibiting ERK activity with a MEK1 inhibitor, U0126, rescued FOXO3a protein levels (Fig. 6F, H) and increased its transcriptional activities (Fig. 6G, I) in HT29 (BRAF^V600E^) and Caco-2-CR cells. The results indicate that there is a decrease in the expression of FOXO3a protein and a reduction in the transcriptional activity of FOXO3a in CRC cells that harbor KRAS/BRAF mutations as well as in CRC cells that have developed resistance to cetuximab treatment. This implies that the downregulation of FOXO3a protein may contribute to resistance to cetuximab. To validate this hypothesis, we overexpressed FOXO3a and evaluated the response to cetuximab in cetuximab-resistant CRC cells. FOXO3a overexpression increased its transcriptional activity and sensitized DLD-1 (KRAS^G13D/-^) cells to cetuximab (Fig. 6J, K, L). Similar effects of FOXO3a overexpression were observed in HT29 (BRAF^V600E^) (Fig. 6M, N, O) and Caco-2-CR cells (Fig. 6P, Q, R). Our findings suggest that the downregulation of FOXO3a and its decreased transcriptional activity contribute to resistance to cetuximab.

## Discussion

In this study, we demonstrate that co-treatment with 3-BP and cetuximab synergistically induces an antiproliferative effect by triggering apoptosis and autophagy-dependent ferroptosis, both in vitro and in vivo. Mechanistically, co-treatment with 3-BP and cetuximab inhibited the phosphorylation and degradation of FOXO3a, thereby rescuing the FOXO3a protein level and its transcriptional activity (as illustrated in Fig. 6S). The upregulation of FOXO3a activates the FOXO3a/AMPKα/pBeclin1 pathway, which promotes ferroptosis by blocking system Xc^−^ activity. Furthermore, this co-treatment also triggers apoptosis through the FOXO3a/PUMA pathway. We provided experimental evidence that co-treatment with cetuximab and 3-BP could be a potential therapeutic strategy to overcome resistance to cetuximab in colorectal cancer.

The KRAS and BRAF genes encode proteins that play a critical role in regulating cell growth and proliferation [24–26]. Mutations in these genes are commonly associated with several types of cancers, including colorectal, lung, and pancreatic cancers [27–31]. KRAS/BRAF mutations can result in the constitutive activation of EGFR signaling through the oncogenic RAS/RAF/MEK/ERK pathway [32, 33]. Activated RAS-ERK signaling can lead to the phosphorylation and degradation of FOXO3a, a transcription factor that plays a role in various cellular processes, including apoptosis [34–36], autophagy [37, 38] and ferroptosis [39], inhibiting its transcriptional activity [19]. Consistent with these findings, our study demonstrated that FOXO3a protein levels are downregulated and its transcriptional activity is inhibited in HCT116 and DLD-1 cells with the KRAS^G13D^ mutation, as well as in RKO cells with the BRAFV^600E^ mutation, compared to their wild-type KRAS/BRAF parent cells. Overexpression of FOXO3a sensitizes DLD-1 (KRAS^G13D/-^) and HT29 (BRAF^V600E^) cells to cetuximab, which is consistent with previous studies showing that knockdown of FOXO3a desensitizes DLD-1 (KRAS^G13D/-^) cells to cetuximab [40]. These findings highlight that KRAS/BRAF mutations lead to the downregulation of FOXO3a and contribute to cetuximab resistance in CRC cells with KRAS/BRAF mutations. Furthermore, we also assessed acquired resistance to cetuximab in Caco-2-CR cells and found that FOXO3a protein levels were downregulated and its transcriptional activity was inhibited. FOXO3a overexpression sensitizes Caco-2-CR cells to cetuximab, indicating that the downregulation of FOXO3a contributes to cetuximab resistance in wild-type KRAF/BRAF CRC cells. In this study, we found that co-treatment with 3-BP and cetuximab inhibited FOXO3a phosphorylation and degradation, increasing its transcriptional activity. This combined treatment shows potential in overcoming cetuximab resistance in colorectal cancer (CRC) cells that carry KRAF/BRAF mutations or have developed acquired resistance.

Our findings suggest that FOXO3a mediates the cell death induced by co-treatment with 3-BP and cetuximab. Our results are consistent with previous studies that have elucidated the role of FOXO3a in autophagy and apoptosis [37, 38, 41–43]. The co-treatment resulted in an upregulation of SLC7A11 expression but a concurrent inhibition of system Xc^−^ activity through the FOXO3a/AMPKα/pBeclin1 pathway. This led to a depletion of GSH and ultimately induced ferroptosis. The upregulation of SLC7A11 expression may act as a negative feedback loop to limit lipid peroxidation.

Ferroptosis was initially characterized as an iron-dependent form of cell death distinct from apoptosis, necrosis, and autophagy [44]. However, recent evidence has challenged this notion and suggests that the autophagic machinery actually contributes to ferroptotic cell death [9, 45]. As a result, ferroptosis is now recognized as a process that can be influenced by and dependent on autophagy. According to previous reports, autophagy can promote ferroptosis through various mechanisms, including regulation of iron metabolism [46], lipid peroxidation [47, 48], organelle homeostasis [49], and cystine depletion [50, 51]. Supporting these findings, we showed that genetic disruption of autophagy by either Beclin1 or ATG5 knockdown blocks 3-BP/cetuximab-induced ferroptosis. These collective findings provide further evidence for the notion that ferroptosis is influenced and dependent on autophagy.

3-BP is considered one of the most promising antitumorigenic agents that suppresses tumor growth and induces cell death by multiple mechanisms and has been shown to display cytotoxic effects in multiple cancers [52–57]. However, the complex nature of the mechanism of toxicity conferred 3-BP not only antitumor activity but also side effects, including hepatotoxicity [58–60]. To overcome this shortcoming, a combination of 3-BP and another drug, which induced a synergistic antitumor effect and decreased 3-BP doses, might overcome these drawbacks. In this study, we showed that co-treatment with 3-BP and cetuximab induced synergistic antiproliferative effects. This synergistic effect decreased the doses of 3-BP, resulting in less undesirable toxicity and side effects but a better anticancer effect in cetuximab-resistant CRC cells.

In conclusion, our results provide evidence that co-treatment with 3-BP and cetuximab induced synergistic antiproliferative effects by triggering apoptosis and autophagy-dependent ferroptosis in CRC cells that carry KRAF/BRAF mutations or have developed acquired resistance. The co-treatment of cetuximab and 3-BP could be a potential therapeutic strategy to overcome resistance to cetuximab in colorectal cancer.

### Supplementary information

Supplementary legend

Supplementary Figure 1

Supplementary Figure 2

Supplementary Figure 3

Supplementary Figure 4

Uncropped western blot

## Data Availability

All data needed to evaluate the conclusions in the paper are presented in the paper. Additional data related to this paper may be requested from the corresponding author.
