# Supplementary material for: 3-Bromopyruvate overcomes cetuximab resistance in human colorectal cancer cells by inducing autophagy-dependent ferroptosis
Source: Cancer Gene Ther. 2023 Aug 9;30(10):1414–25. doi: 10.1038/s41417-023-00648-5 (PMC10581902; doi:10.1038/s41417-023-00648-5)
Supplement: Supplementary file 1 — Supplementary legend [file 41417_2023_648_MOESM1_ESM.docx]

Supplementary Figure 1. (**A**) The protein level of BRAF in HT29 (BRAF^V600E^) transfected with or without shRNA targeting BRAF. (**B**) The chemical structure of 3-BP. (**C, D**) The antiproliferation effect of combined 10 μg/ml Cetuximab with 3-BP of the indicated concentration for four days in DLD-1 (KRAS^G13D/-^) (**C**)and Caco-2-CR (**D**). (**E**) The antiproliferation effect of 3-BP, Cetuximab, or their combination at a fixed proportion in HT29 (BRAF^V600E^). (**F**) The Fa-CI plot of the co-treatment in HT29(BRAF^V600E^). The CI value<1 is the definition of synergism. Data are expressed as mean ± SD, n = 3 biological replicates.

Supplementary Figure 2. (**A**) The effect of the combination of different cell death inhibitors on the anticancer activity of the co-treatment of 3-BP (5 μM) and Cetuximab (10 μg/ml) in Caco-2-CR, DLD-1(KRAS^G13D/-^) and HT29(BRAF^V600E^). (**B**) The FerroOrange visualized Fe^2+^ level in HT29 (BRAF^V600E^) or Caco-2-CR cells following the treatment of 3-BP (5 μM), Cetuximab (10 μg/ml) or their combination for four days. (**C**) ATG5 (**Left**) or Beclin1 (**Right**) knockdown in DLD-1(KRAS^G13D/-^) cells, HT29(BRAF^V600E^) cells, or Caco-2-CR cells. (**D-H**) The effect of ATG5 or Beclin1 knockdown on the Fe2+ accumulation (**D**), glutamate release inhibition (**E**), GSH depletion (**F**), cell viability inhibition (**G**), and MDA production (**H**) induced by the co-treatment of 3-BP (5 μM) and Cetuximab (10 μg/ml). Data are expressed as mean ± SD, n = 3 biological replicates.

Supplementary Figure 3. (**A**) The protein level of FOXO3a and PUMA following the co-treatment of 3-BP (5 μM) and Cetuximab (10 μg/ml) four days with or without FOXO3a knockdown. (**B**) The protein level of PUMA and cleaved caspase -3 following the co-treatment of 3-BP (5 μM) and Cetuximab (10 μg/ml) four days with or without PUMA knockout. (**C**) Flow cytometry analysis of the apoptotic cells following the co-treatment of 3-BP (5 μM) and Cetuximab (10 μg/ml) four days with or without FOXO3a knockdown. (**D**) The FOXO3a, LC3b, and p62 protein levels following the co-treatment of 3-BP (5 μM) and Cetuximab (10 μg/ml) four days with or without FOXO3a knockdown. Data are expressed as mean ± SD, n = 3 biological replicates. ns: not significant.

Supplementary Figure 4. (**A**) The xenograft nude mouse models were established using HT29 (BRAF^V600E^) cells with or without FOXO3a knockdown. The body weights, tumor volumes, and tumor weights were evaluated following the treatment of PBS (0.2 ml), or a combination of 3-BP (2 mg/kg/day, dissolved in 0.2 ml PBS) and Cetuximab (25 mg/kg/day, dissolved in 0.2 ml PBS). (**B**) Immunoblotting analyzed protein levels of LC3b, p62, Cleaved Caspase-3, and FOXO3a in tumor xenografts. (**C-E**) The effect of FOXO3a knockdown on the co-treatment-induced MDA production (**C**), Fe^2+^ accumulation (**D**), and GSH depletion (**E**) in tumor xenografts were evaluated. (**F**) The xenograft nude mouse models were established using Caco-2-CR cells with or without FOXO3a knockdown. The body weights, tumor volumes, and tumor weights were evaluated following the treatment of PBS (0.2 ml), or a combination of 3-BP (2 mg/kg/day, dissolved in 0.2 ml PBS) and Cetuximab (25 mg/kg/day, dissolved in 0.2 ml PBS). (**B**) Immunoblotting analyzed protein levels of LC3b, p62, Cleaved Caspase-3, and FOXO3a in tumor xenografts. (**C-E**) The effect of FOXO3a knockdown on the co-treatment-induced MDA production (**C**), Fe^2+^ accumulation (**D**), and GSH depletion (**E**) in tumor xenografts were evaluated. Data are expressed as mean ± SD, n = 5 biological replicates. ns: not significant.
