# Supplementary figures and images for: 3-Bromopyruvate overcomes cetuximab resistance in human colorectal cancer cells by inducing autophagy-dependent ferroptosis
Source: Cancer Gene Ther. 2023 Aug 9;30(10):1414–25. doi: 10.1038/s41417-023-00648-5 (PMC10581902; doi:10.1038/s41417-023-00648-5)

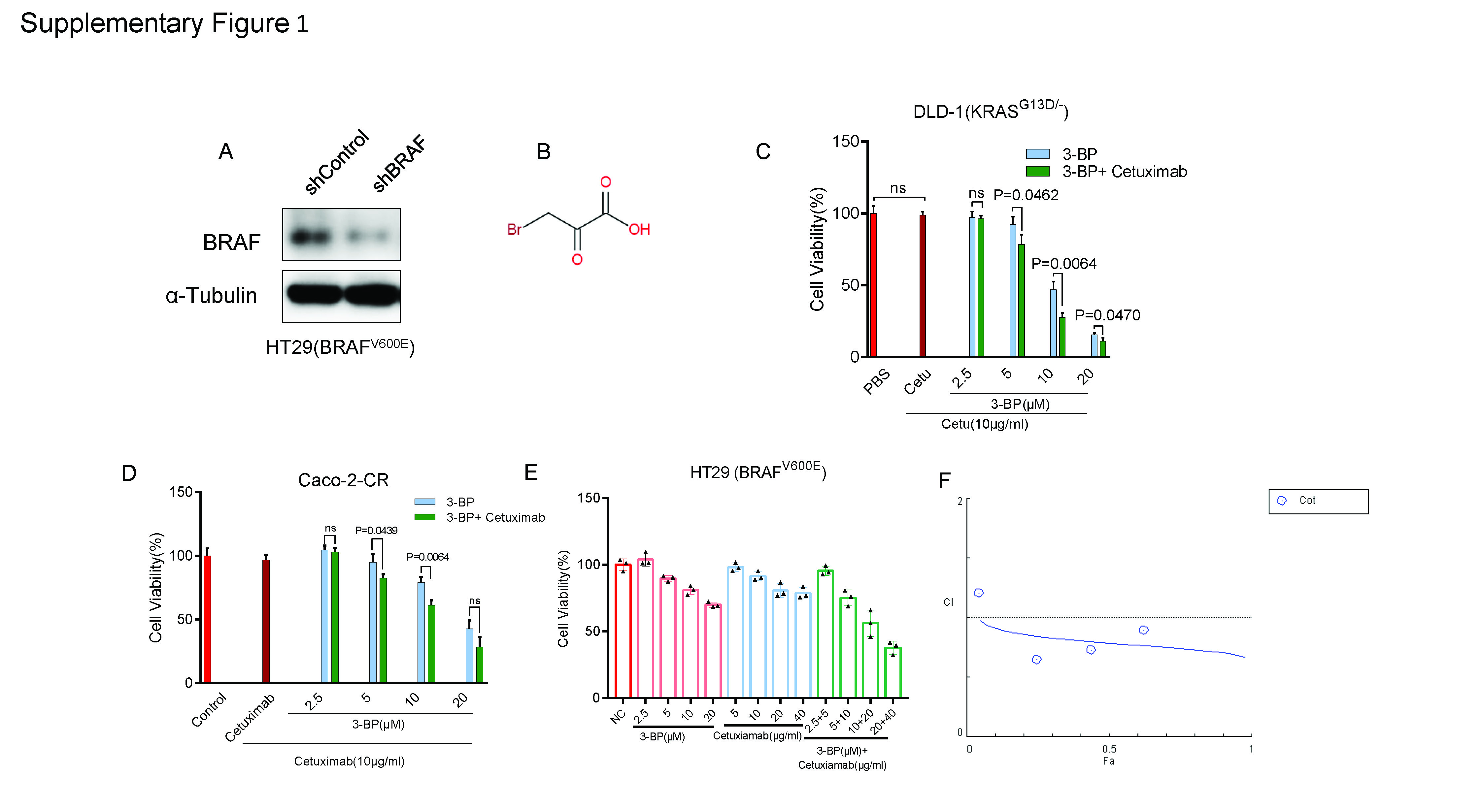

Supplement: Supplementary file 2 — Supplementary Figure 1 [file 41417_2023_648_MOESM2_ESM.jpg]

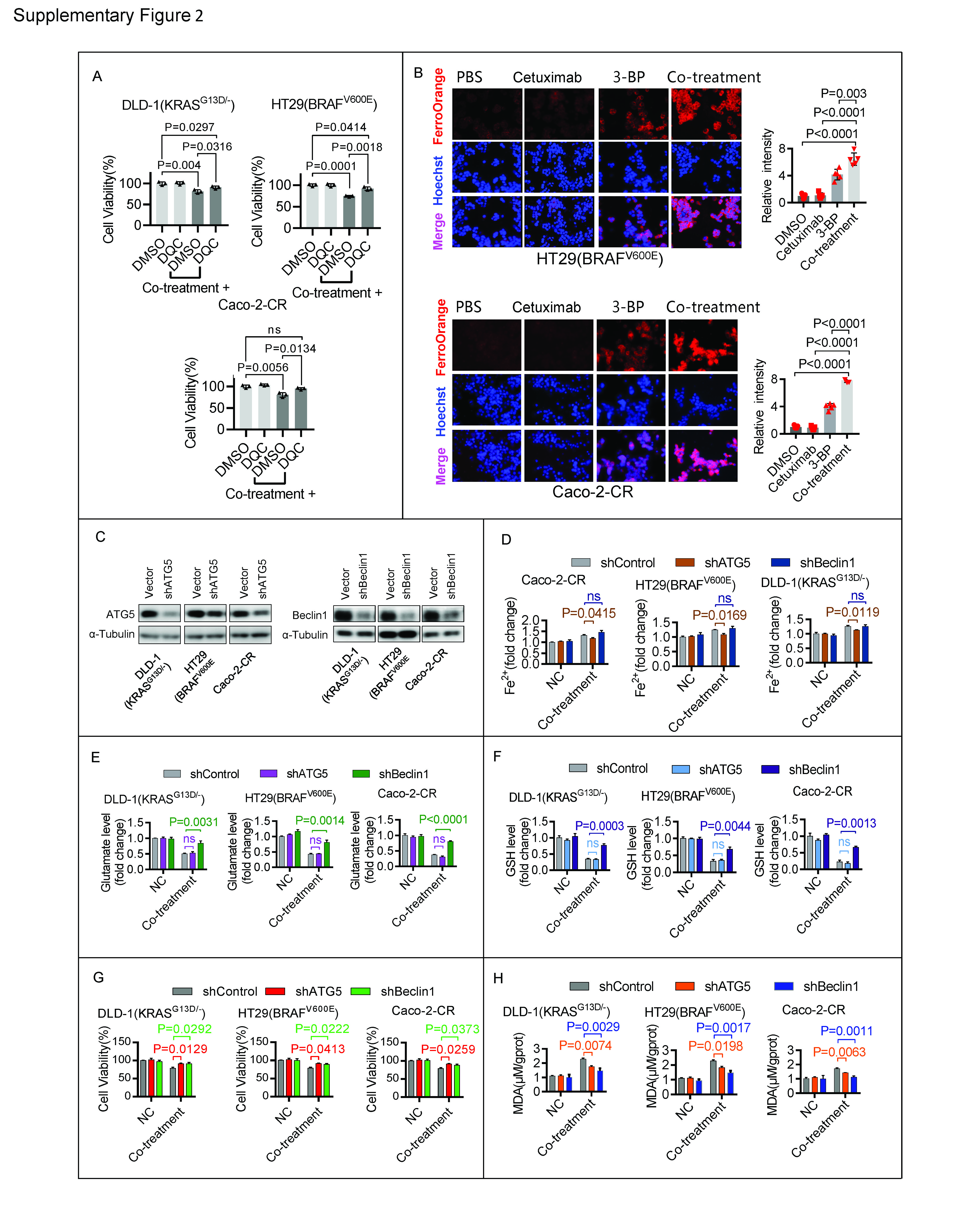

Supplement: Supplementary file 3 — Supplementary Figure 2 [file 41417_2023_648_MOESM3_ESM.jpg]

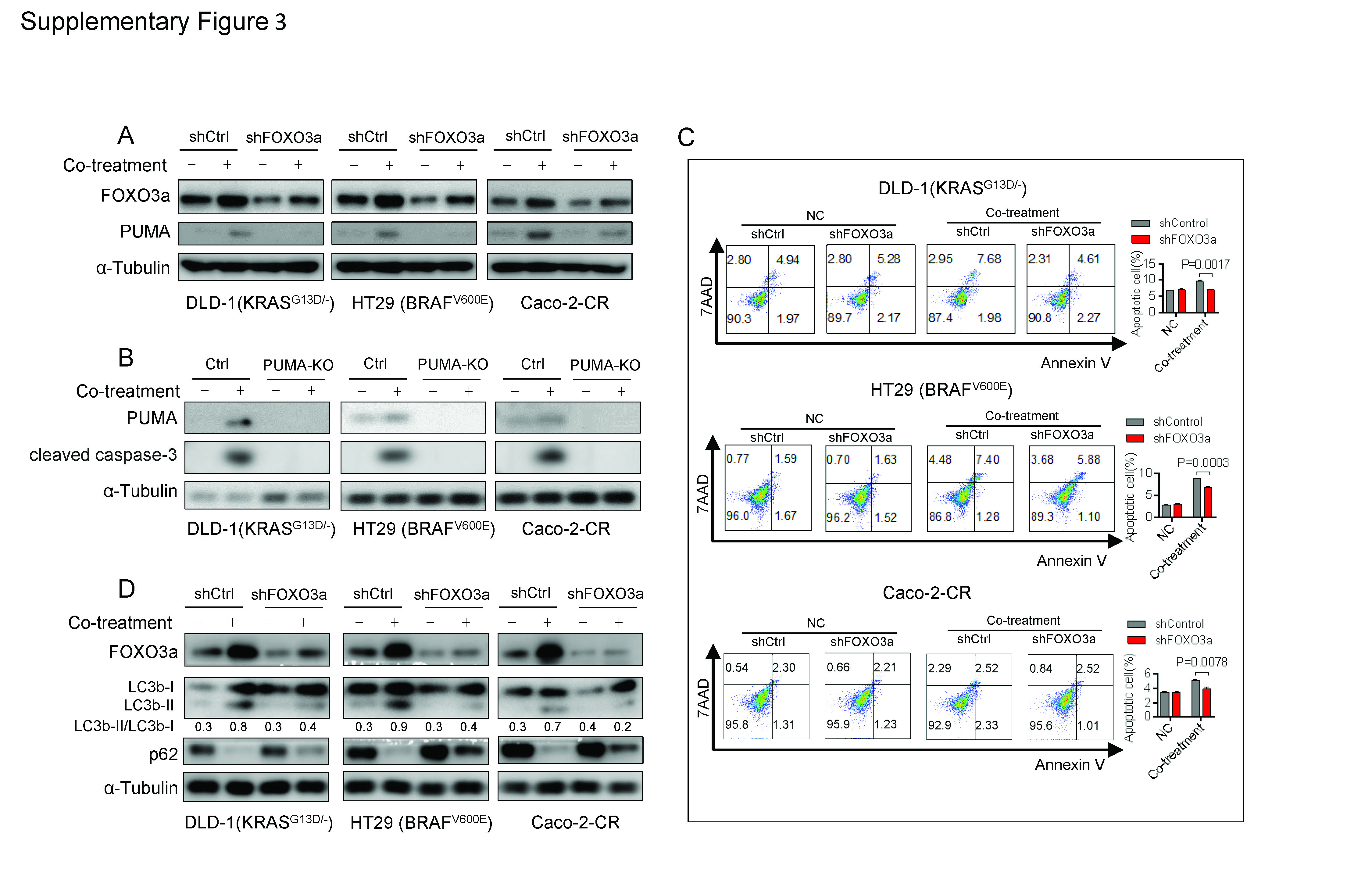

Supplement: Supplementary file 4 — Supplementary Figure 3 [file 41417_2023_648_MOESM4_ESM.jpg]

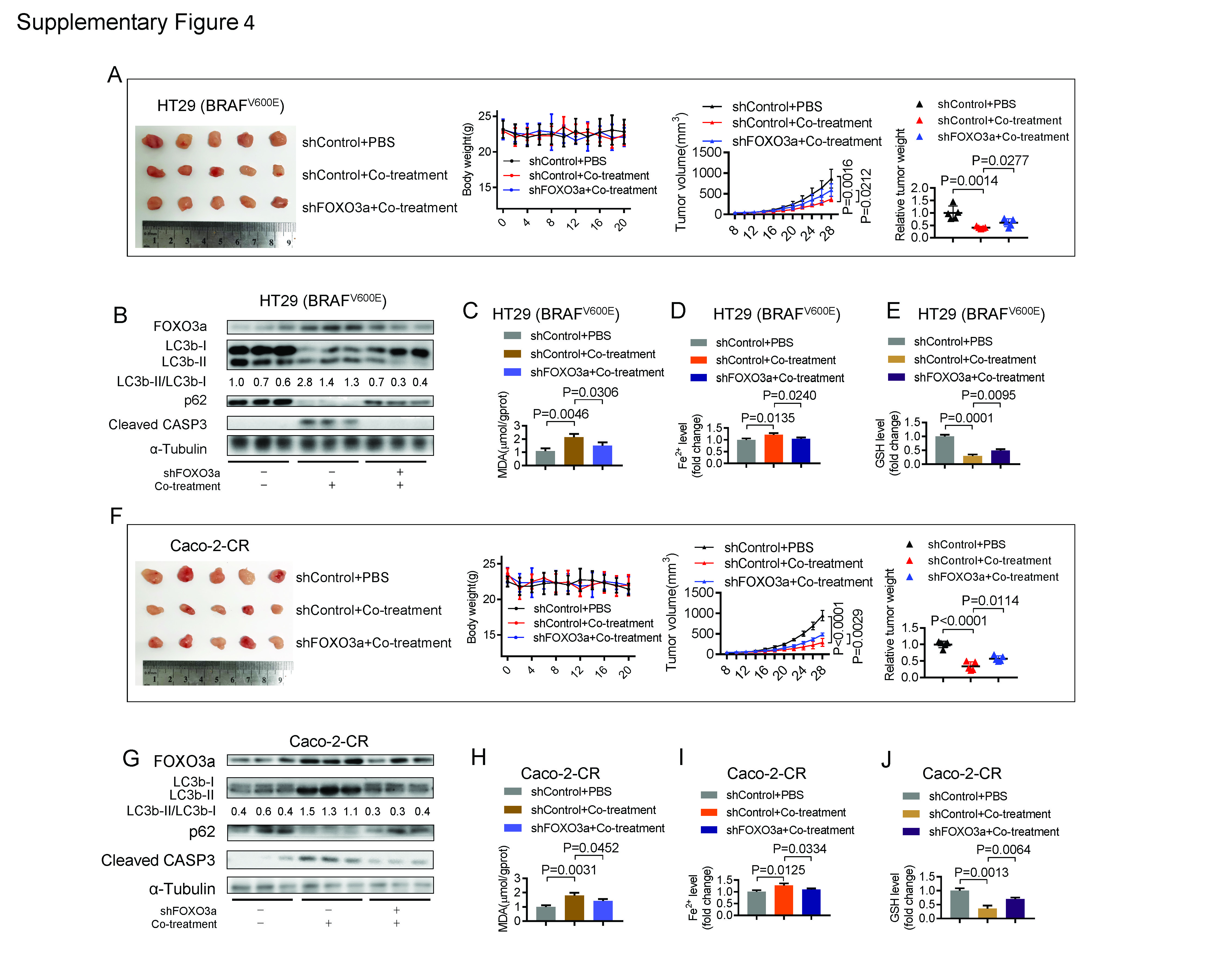

Supplement: Supplementary file 5 — Supplementary Figure 4 [file 41417_2023_648_MOESM5_ESM.jpg]
