# Supplementary material for: 3-Bromopyruvate overcomes cetuximab resistance in human colorectal cancer cells by inducing autophagy-dependent ferroptosis
Source: Cancer Gene Ther. 2023 Aug 9;30(10):1414–25. doi: 10.1038/s41417-023-00648-5 (PMC10581902; doi:10.1038/s41417-023-00648-5)

Figure 2B

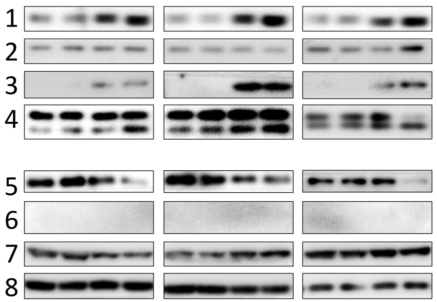
1
2
3
4
5
6
7
8

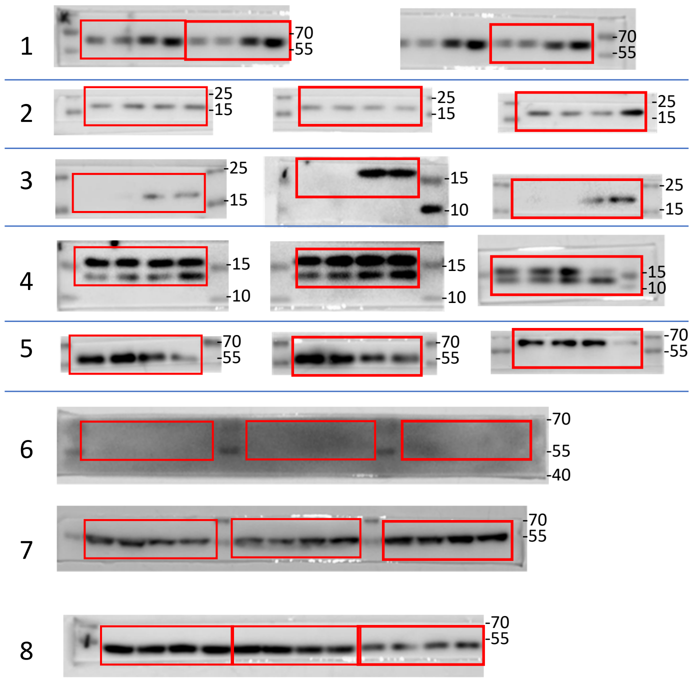
1
-70
-55
2
-25
-15
3
-25
-15
-10
4
-15
-10
5
-70
-55
6
-70
-55
-40
7
-70
-55
8
-70
-55

Figure 3A

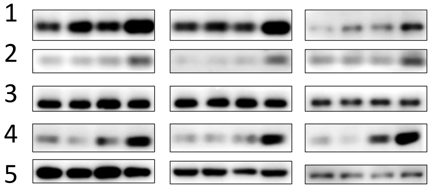
1
2
3
4
5

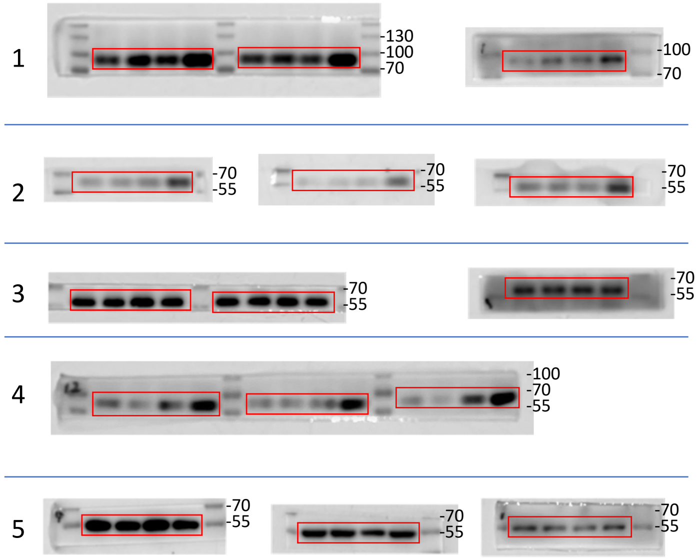
1
-130
-100
-70
-100
-70
2
-70
-55
-70
-55
-70
-55
3
-70
-55
-70
-55
4
-100
-70
-55
5
-70
-55
-70
-55
-70
-55

Figure 3B

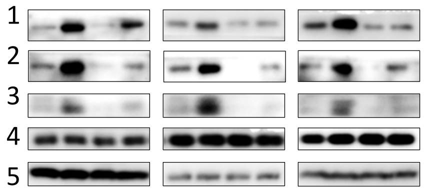

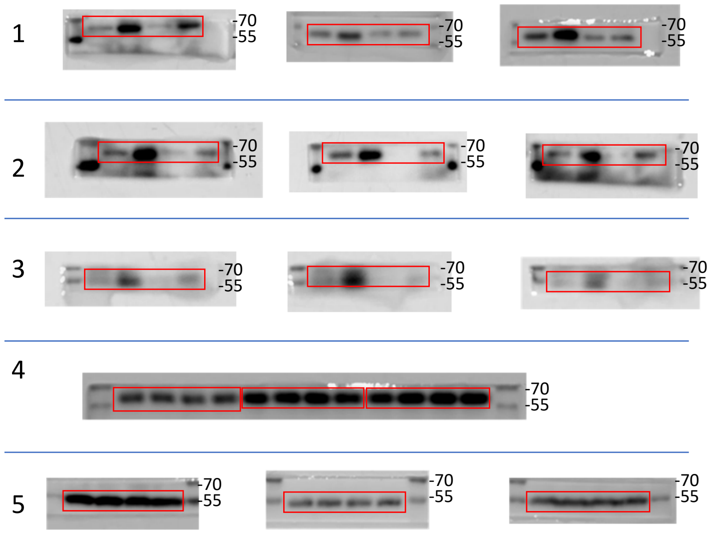

Figure 3G

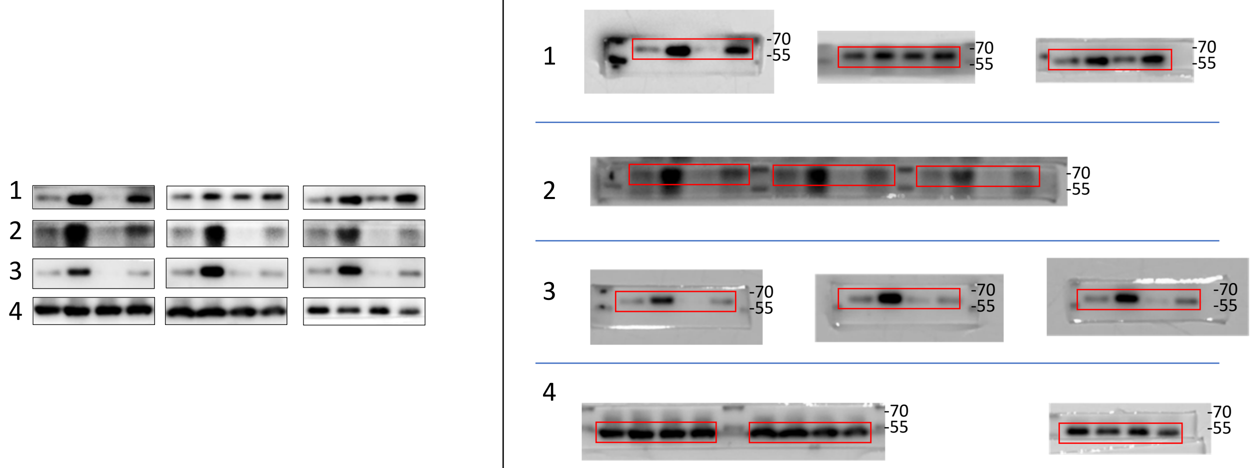

Figure 3L

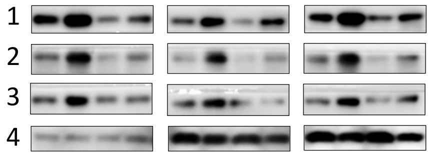

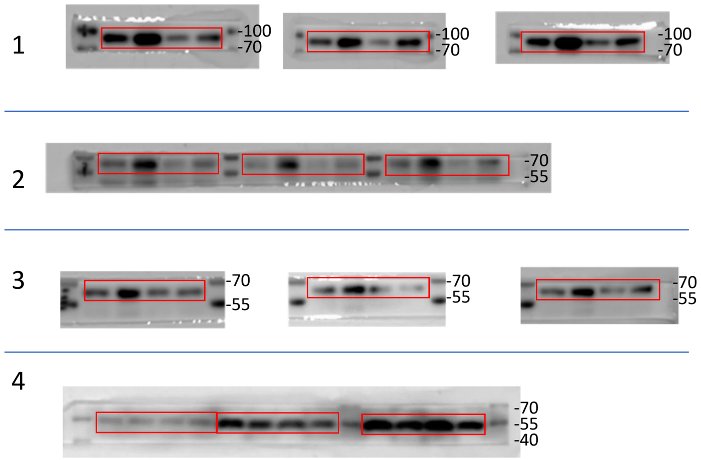

Figure 4A

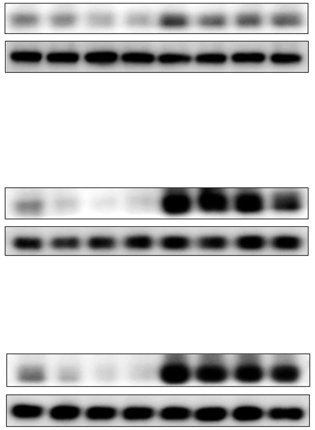

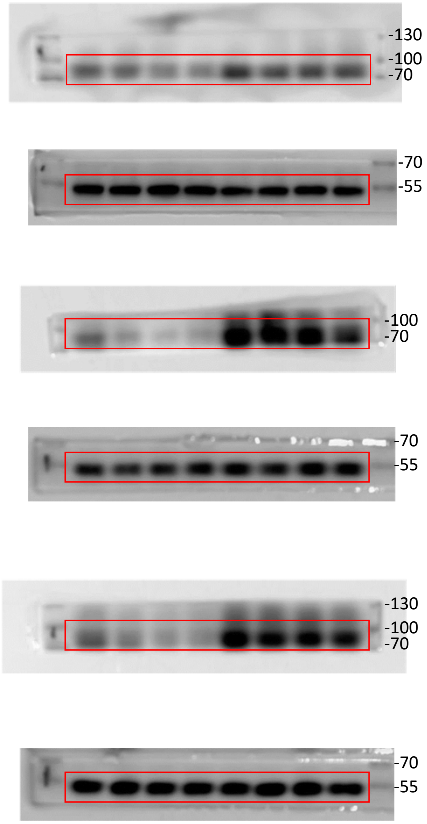

Figure 4B

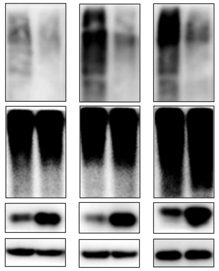

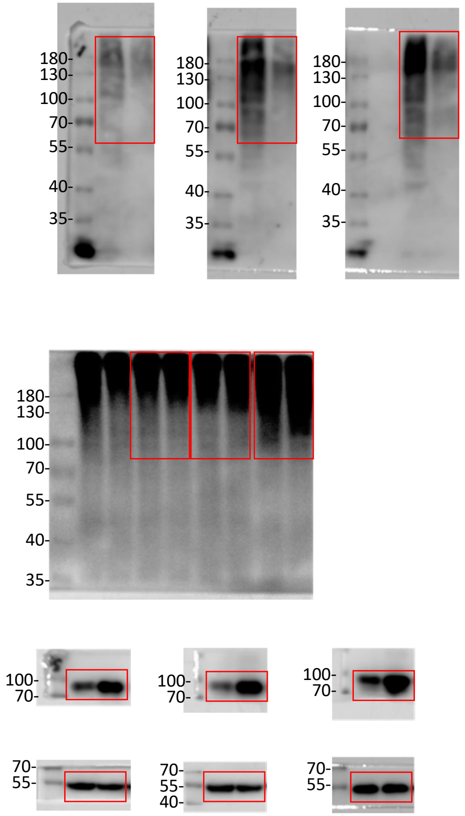

Figure 4C

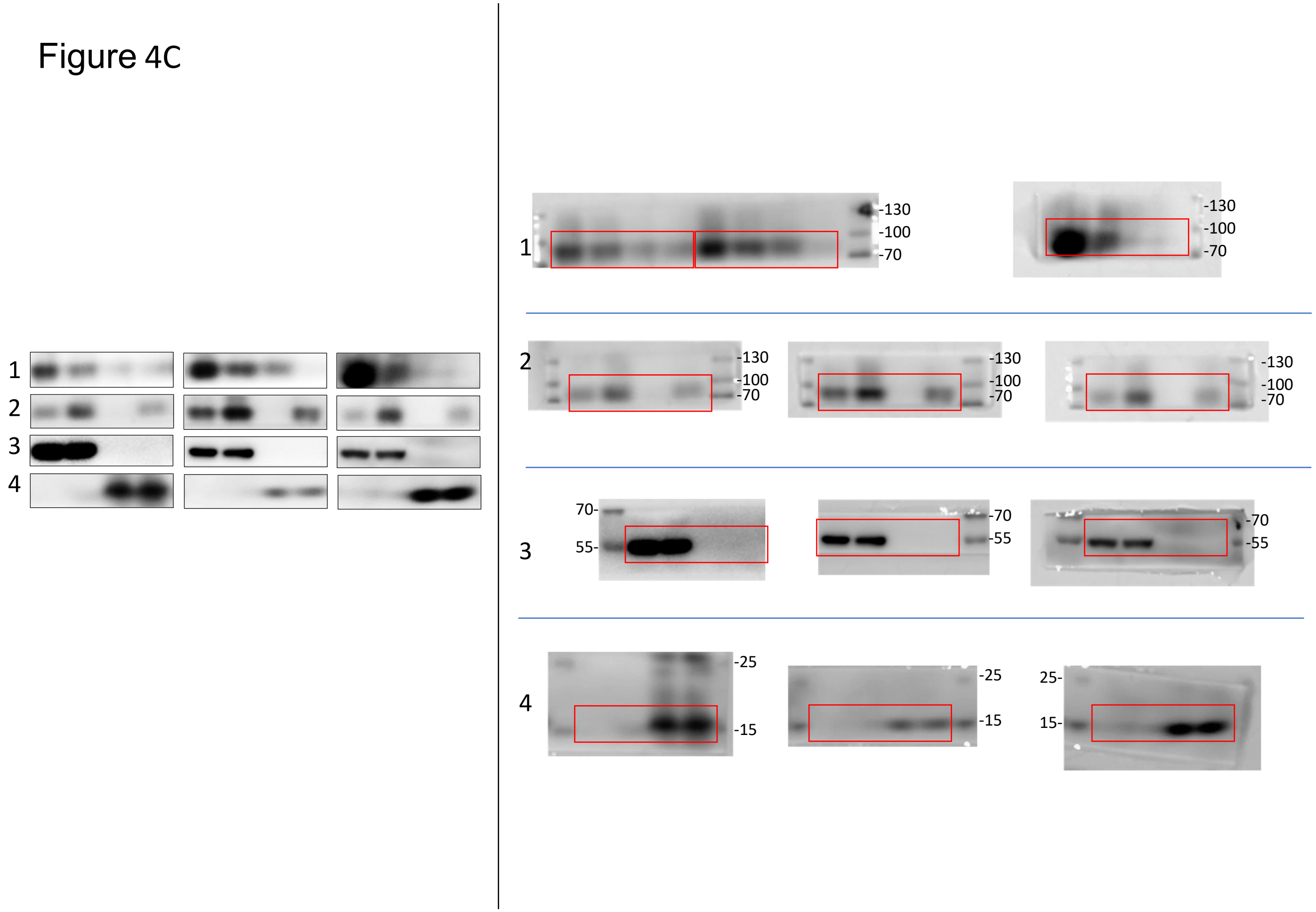
1
2
3
4
1
-130
-100
-70
-130
-100
-70
2
-130
-100
-70
-130
-100
-70
-130
-100
-70
3
70-
55-
-70
-55
-70
-55
4
-25
-15
-25
-15
25-
15-

Figure 4D

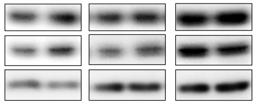

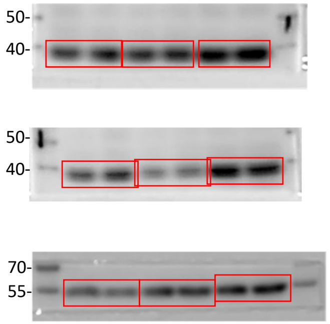

Figure 5A

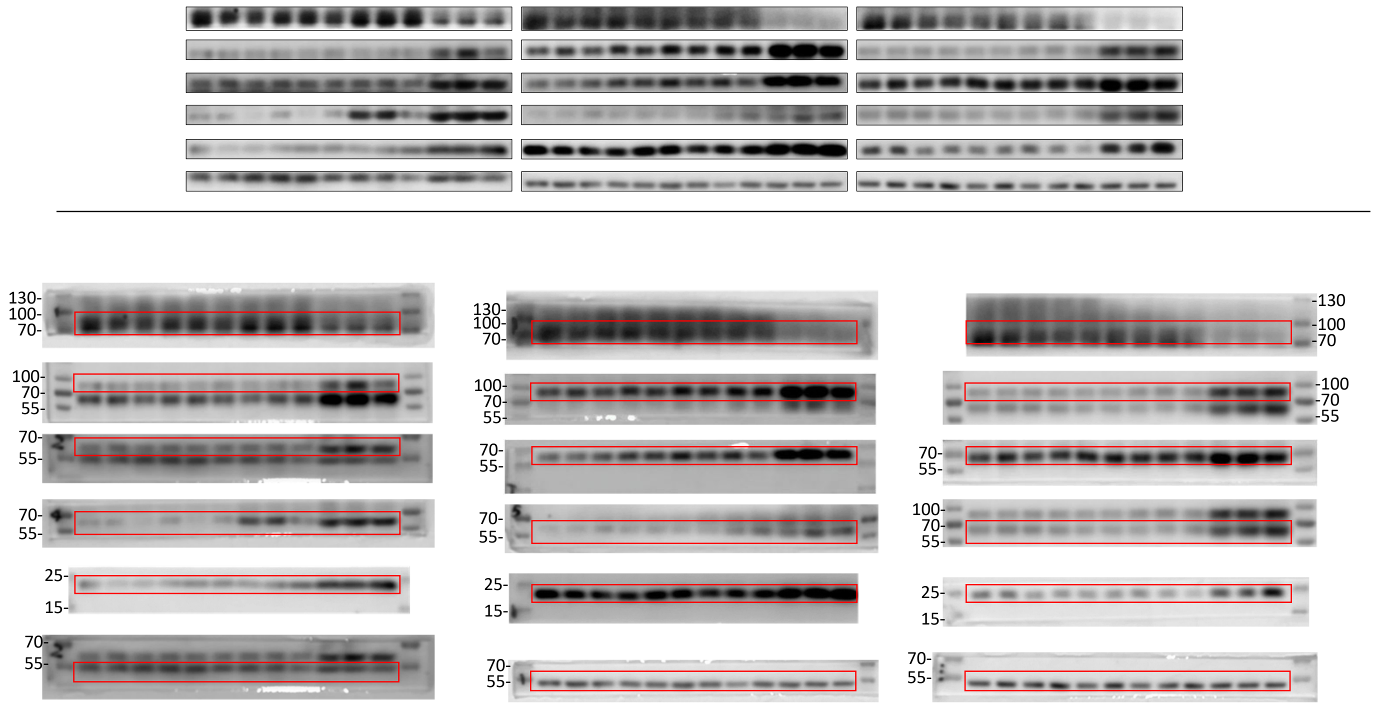
130-
100-
70-
100-
70-
55-
70-
55-
70-
55-
25-
15-
70-
55-
130-
100-
70-
100-
70-
55-
70-
55-
70-
55-
25-
15-
70-
55-
-130
-100
-70
-100
-70
-55
70-
55-
100-
70-
55-
25-
15-
70-
55-

Figure 5C

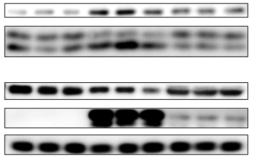

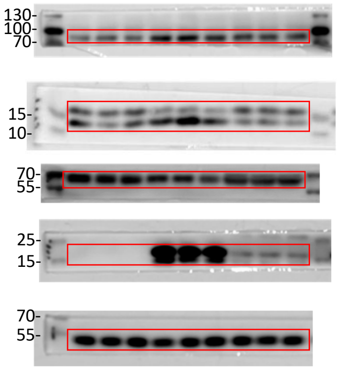

Figure 6A

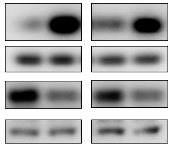

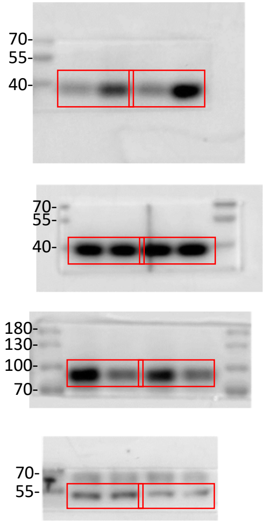

Figure 6B

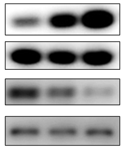

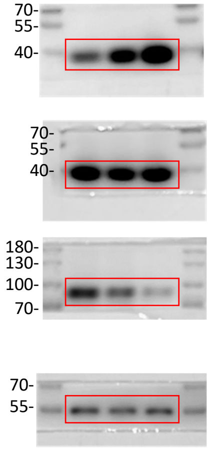

Figure 6C

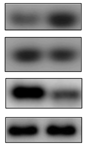

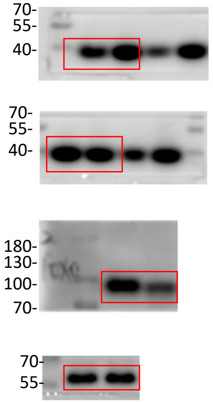

Figure 6D

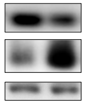

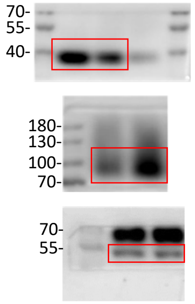

Figure 6F

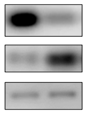

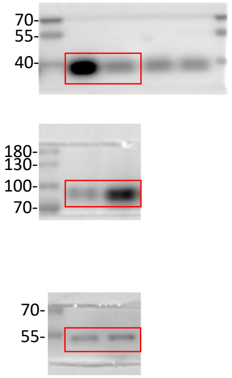

Figure 6H

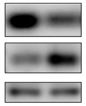

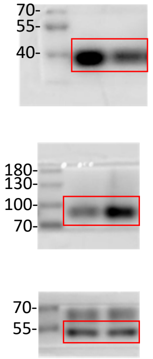

Figure 6J

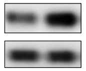

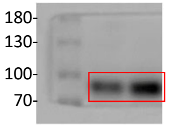

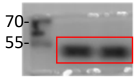

Figure 6M

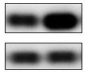

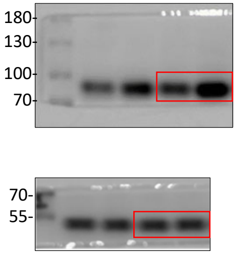

Figure 6P

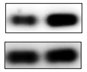

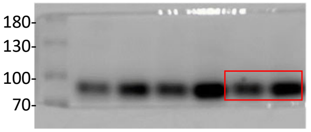

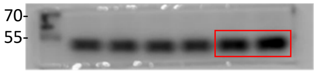

Supplementary Figure 1A

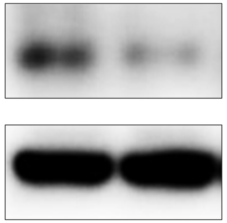

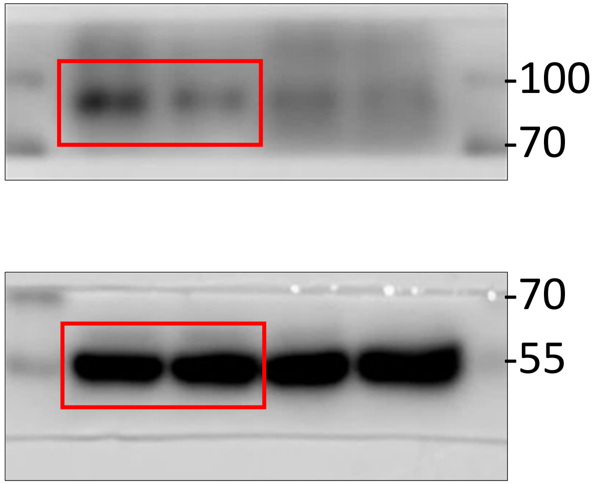

Supplementary Figure 2C

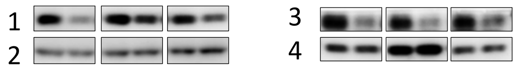

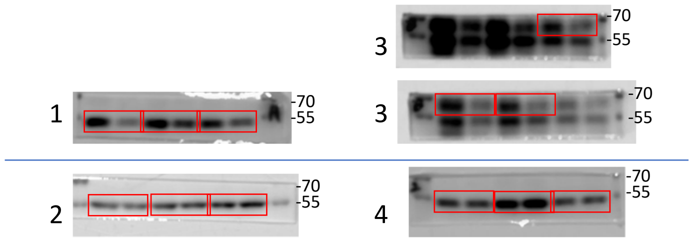

Supplementary Figure 3A

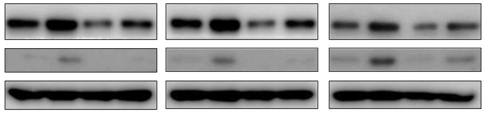

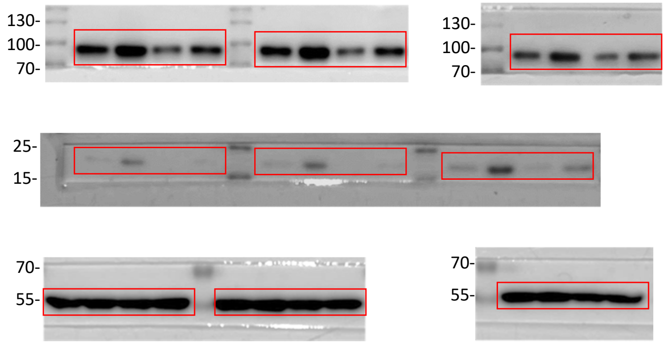

Supplementary Figure 3B

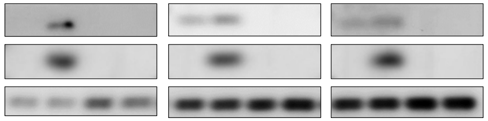

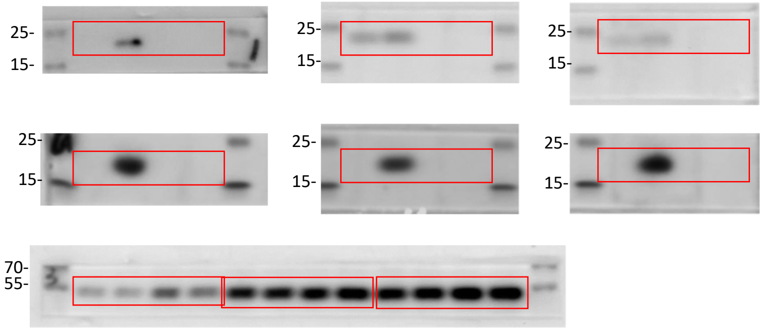

Supplementary Figure 3D

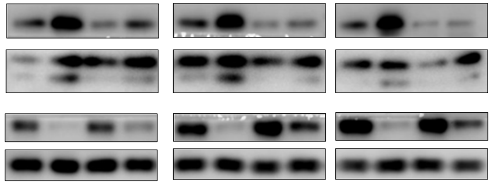

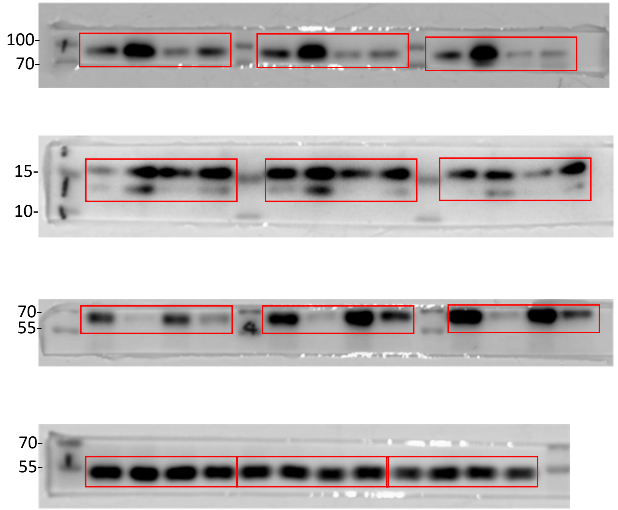

Supplementary Figure 4B

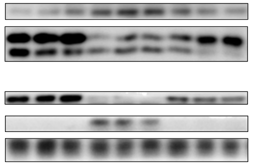

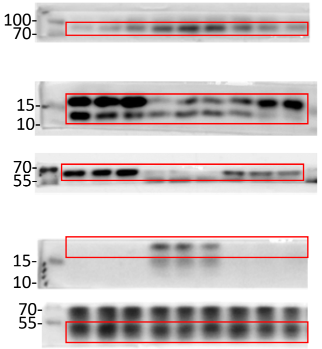

Supplementary Figure 4G

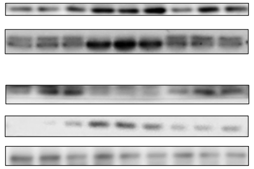

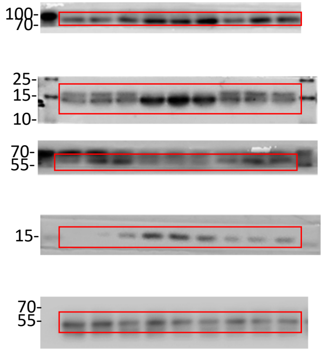

Supplement: Supplementary file 6 — Uncropped western blot [file 41417_2023_648_MOESM6_ESM.pdf]
